# Increasing Knowledge and Self-Efficacy on Differences in Sex Development (DSD): A Team-Based Learning Activity for Pediatric Residents

**DOI:** 10.15766/mep_2374-8265.11105

**Published:** 2021-02-23

**Authors:** Anshu Gupta, Kelly Lockeman, Cherie Edwards

**Affiliations:** 1 Associate Professor, Department of Pediatrics, Virginia Commonwealth University School of Medicine; 2 Associate Professor, Office of Assessment, Evaluation, and Scholarship, Virginia Commonwealth University School of Medicine; 3 Assistant Professor, Office of Assessment, Evaluation, and Scholarship, Virginia Commonwealth University School of Medicine

**Keywords:** Differences in Sex Development, Pediatric Residents, Team-Based Learning, Diversity, Inclusion, Health Equity

## Abstract

**Introduction:**

Differences in sex development (DSD) are a heterogenous group of conditions estimated to affect 1 in 4500 infants. A paradigm shift has occurred in societal and cultural acceptance of variant gender outcomes along with increased awareness around diagnostic uncertainty inherent to DSD. Lack of provider knowledge in evaluation of DSD and/or awareness of evolving paradigms relevant to care for patients with DSD can accentuate barriers to access optimal care for this already vulnerable population.

**Methods:**

To address this unmet need, we used Kern's six-step framework and piloted a team-based learning (TBL) activity for pediatric residents and medical students (36 learners). This included preactivity reading, an 11-item self-efficacy survey around treatment of patients with DSD at the beginning of the TBL, and a seven-question individual readiness assurance test (RAT). Mixed teams of five to seven learners completed the RAT in small groups followed by large-group discussion. An application exercise followed with two cases focused on initial evaluation of a newborn/child with suspected DSD and an older child with suspected DSD. At the conclusion, learners repeated the self-efficacy measure and answered several evaluation questions.

**Results:**

Individual RAT scores had a mean of 59%, while groups scored with a mean of 82%. Mean self-efficacy scores also increased significantly from 2.4 to 3.4 on a 5-point scale. Of learners, 80% agreed or strongly agreed that the activity was effective for improving DSD skills and knowledge.

**Discussion:**

TBL is a valuable educational strategy to enhance knowledge and self-efficacy of DSD care for general pediatricians.

## Educational Objectives

By end of this activity, learners will be able to:

1. Describe common clinical presentations of differences in sex development (DSD).
2. Apply principles of sex differentiation/development to evaluate a child with suspected DSD through history and physical exam.
3. Apply principles of sex differentiation/development to order initial laboratory testing in a child with suspected DSD.
4. Summarize the indications for emergency medical evaluation and subspecialist evaluation in a child with suspected DSD.
5. Explore principles for counseling children (when applicable) and family of a child with suspected DSD and the role of various disciplines in the creation of a DSD management plan.

## Introduction

Differences in sex development (DSD) are a heterogenous group of conditions estimated to affect 1 in 4,500 infants. The foundational knowledge of individual factors that contribute to atypical sex development such as chromosomes, genes, anatomy, and hormones is needed by clinicians caring for an individual born with DSD. Additionally, diagnostic and treatment uncertainty for gender and surgery outcomes is inherent to many of DSD conditions, and there has been a paradigm shift in societal and cultural acceptance of variant gender outcomes in the past decade. This has led to controversies regarding appropriate timing of gender assignment and extent of surgery needed specially in newborns with atypical genitalia related to DSD.^1^ In keeping with current recommendations for terminology for DSD, we use the term ‘atypical’ in place of ‘ambiguous’ except when used in the context of citing a source.

Gaps in knowledge (foundational and of current social context including controversies around appropriate care^2^) and comfort of health care providers can make children and families affected by DSD vulnerable to disparities in accessing and receiving care across the developmental continuum. Consequently, the AAMC has recommended development of curricula specifically designed to address these gaps.^3^ Similarly, the American Board of Pediatrics requires all board-certified general pediatric providers to know the diagnosis and management of patients with ambiguous genitalia/DSD.^4^ However, there is an absence of research examining how knowledge about these topics can be imparted to learners and the degree to which pediatric residency trainees are comfortable in this area of medical care. The absence of literature and educational content focused on provider knowledge and self-efficacy around DSD illustrates a need to identify and engage in educational techniques that assist in developing the competencies and practical skills necessary to provide care to these patients. Team-based learning (TBL), an approach that is widely employed in medical education, offers a viable option for such training.

TBL is a learner-centered instructional strategy using small groups to actively improve knowledge, application, and team-building skills.^5^ Across numerous settings, TBL has proven to be equivalent if not superior to didactic learning in enhancing content knowledge. First applied in the undergraduate medical education setting, TBL is also being used at the residency level, with demonstrated positive effects on learner engagement, satisfaction, and most importantly, knowledge.^5,6^ With TBL, learners master course content and apply concepts to solve problems that they will likely face in practice. Care for patients with DSD involves complex issues that require decision making as a team, a process that is also integral to TBL.^5,7^ As such, TBL may be an important teaching method to improve resident education of DSD in children.

We designed and piloted an activity that incorporated DSD-focused content into the TBL instructional approach, and we measured the self-efficacy of learners prior to and after the activity. We hypothesized that participating in this interactive learning strategy would improve pediatric residents’ knowledge and self-efficacy in approach, diagnosis, and management of patients with DSD. This study examined both quantitative and qualitative evaluation data collected as part of this pilot.

## Methods

The Virginia Commonwealth University School of Medicine pediatric residency program has dedicated time each week when all learners, regardless of training year, participate in didactic activities. Faculty members and fellows from general pediatrics and subspecialty disciplines deliver content that rotates on a 3-year cycle so that learners are exposed to each topic at least once during their residency program, and the sequence of topics varies each year. Typically, the didactic is a 1-hour lecture with PowerPoint slides. In an effort to engage learners more actively around the complicated topic of managing DSDs, we used Kern's six-step curriculum framework^8^ to develop our TBL activity.

### Advance Preparation

One week prior to the scheduled activity, we sent learners an email advising them that their scheduled didactic would be a TBL activity. The email included instructions for reading two articles^9,10^ pertaining to DSD in preparation for the activity. McCann-Crosby's^9^ article on the evaluation and management of ambiguous genitalia in newborns was published by the American Academy of Pediatrics in *Pediatrics in Review*, a standard reference used in teaching pediatric residents. It addressed: (1) typical sexual development, (2) classification of DSD, and (3) initial approach to a child with ambiguous genitalia. The article by Romao, et al.^10^ discussed the multidisciplinary and controversial aspects of management of DSD, including gender assignment and surgery, and was published by *Pediatric Clinics of North America*, another widely used reference by pediatricians.

### Team Formation

Through random selection, we organized pediatric residents at all levels and medical students into mixed teams of five to seven participants. We gave each team a folder containing all of the materials needed for the learning activities (Appendix A). They began by completing a brief pretest survey (Appendix B) to measure self-efficacy of learners around treatment of patients with DSD. This measure is described in the evaluation strategy below. Then the instructor used the PowerPoint slides (Appendix C) to give a brief introduction to the concept of TBL and an outline of the activity. This introduction was critical for graduate medical learners since TBL is fundamentally different from traditional lectures. Achievement of learning objectives through TBL requires learners to understand the rationale and process by which TBL is conducted (e.g., role of instructor, role of individual members, and role of team).

### Readiness Assurance Process and Immediate Feedback

Following the formation of teams and introduction of the TBL technique, individual learners completed a seven-question readiness assurance test (RAT) (Appendix D) to assess knowledge and mastery of the required prereading. The use of reference materials was not permitted during the readiness assurance process. Once the learners completed the preliminary testing, members collaborated within their teams to complete the same RAT.

Team members received immediate feedback during team completion of the group RAT (gRAT) by way of an immediate feedback assessment technique (IF/AT) form. When all teams had completed the RAT and the IF/AT process, we reviewed test items one by one as a large group using the PowerPoint slides (Appendix C) and the facilitator version of the RAT (Appendix E) as a guide. We gave learners the opportunity to ask clarifying questions or submit an appeal in writing for any test items that were perceived as unclear.

### Team Application Activities

We used the PowerPoint slides (Appendix C) and the facilitator version of the activity (Appendix F) to facilitate the team application activity (Appendix G), which consisted of two case vignettes. Case 1 presented the family of a newborn, and case 2 the family of an older child with suspected DSD. We designed both cases around the primary educational objectives described above.

We provided each team with a copy of the adrenal enzyme pathway (Appendix H) and Prader scale (Appendix I) during the application activities since these references may not be feasible to commit to memory. We made no mention about restrictions on using other resources, so learners were able to search for and consult additional reference materials during the application exercises if they wished. Each team worked stepwise and answered corresponding multiple-choice questions for each case. Individual teams discussed questions first within their group to achieve consensus on the team's response; we then prompted teams, question by question, to simultaneously report their selected answer to the larger group via use of color-coded cards, at which time we gave them the opportunity to debate, defend, and appraise their answers. All teams completed case 1 and participated in the simultaneous large-group call-out before moving to case 2. Upon completion of the group application exercises for both cases, we provided a short summary of the activity to reinforce important learning points. We gave learners time to reflect on the day's activity and provide feedback through a brief evaluation survey. This process included the opportunity to appeal questions or answer choices the learners felt were incorrect and to provide a citation for their appeal. We did not assign grades for any of the above activities.

### Facilitation Schema

The facilitation schema is located in Table 1.

**Table 1.**
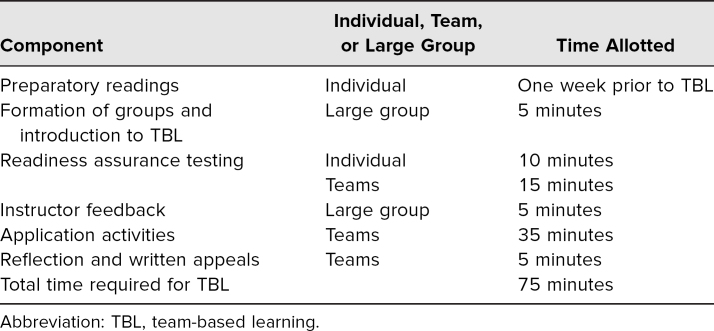
Facilitation Schema

### Evaluation Strategy

#### RAT

We collected individual RAT (iRAT) responses on a paper response form (Appendix B) where a grader later marked whether the response was correct or incorrect. Scores were calculated as the percentage of correct responses out of seven items. Teams recorded gRAT responses on an IF/AT card. Scores for the gRAT were calculated as the total item score out of seven items. Item scores were based on how many attempts a group made before they selected the correct response. A correct response on the first attempt received a score of 1 (3/3); correct responses on the second attempt received a 2/3; on the third attempt, they received a 1/3; and the fourth attempt was a 0. We calculated descriptive statistics to compare the mean iRAT score for each group with the gRAT score.

#### Evaluation survey

We developed a brief self-assessment survey (Appendix B) to measure self-efficacy of learners around treatment of patients with DSD based on AAMC guidelines and evidence-based practice.^3^ The survey consisted of 11 items that were rated on a 5-point scale (1 = *not at all confident in my knowledge/ability*, 5 = *completely confident in my knowledge/ability*). We used the mean item response as an overall measure of self-efficacy. Participants completed the survey before beginning the TBL activity (pretest) and again at the conclusion of the session (posttest). Demographic items on the pretest survey asked participants to disclose their year of training, how many hours of training they had received on DSDs in medical school, and how many times they had seen patients with DSDs. On the posttest, we asked participants to respond on a scale of 0 (*No way!*) to 10 (*Absolutely!*) if they would like to repeat the TBL activity again next year. We also asked about their biggest takeaway from the TBL activity and what they would like to be added given the time allotted.

## Results

### Quantitative

A total of 36 learners participated in the activity. Pediatric residents from PGY 1–3 comprised the majority of the learners (*n* = 28, 78%), but a small number of medical students in their pediatric clerkship (*n* = 6, 17%) also participated. Two learners (6%) did not identify their year of training. There were five groups, and each group included participants at different levels of training. Only seven of the learners (20%) had previously received more than 2 hours of education on DSDs in medical school, and only one (3%) had received DSD education for a personal reason (self or immediate family member). Fifteen (42%) indicated that they had never seen a patient with DSD.

iRAT scores for all 36 participants ranged from 14% to 100%, with a mean of 59% (*SD* = 22%). gRAT scores had a mean of 82% (*SD* = 11%) and ranged from 71% to 100%. gRAT scores were available for only four of the five teams because the IF/AT card for one team was lost following the activity. gRAT scores and iRAT mean scores for each group are displayed in the Figure. The frequency and distribution of incorrect responses on the iRAT were higher for questions around medical history, physical findings, and initial testing, and was notable on the gRAT. For example, only 39% learners chose the correct response for question 3 on pertinent medical history when evaluating a child with genital ambiguity, and only 33% selected the preferred response for question 4 on laboratory testing for a child with given atypical genital physical findings. We did not find a significant difference in iRAT scores between medical students and residents.

**Figure.**
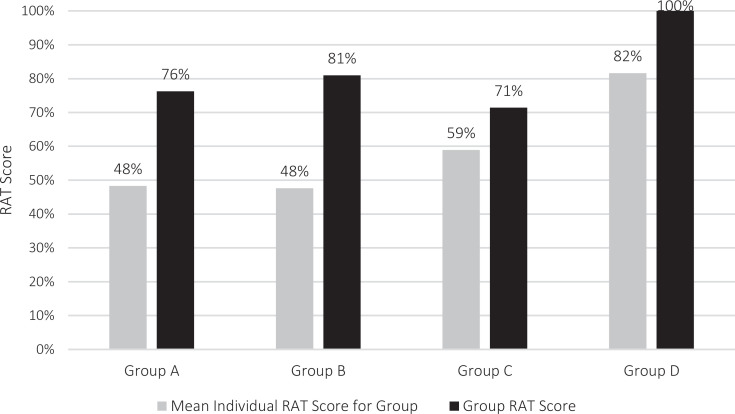
Mean individual and group readiness assurance test (RAT) scores for each group.

Thirty-five participants (97%) completed both the pretest and posttest self-efficacy surveys. The overall item mean at pretest was 2.4 (*SD* = 0.8) on a 5-point scale, while the posttest mean was 3.4 (*SD* = 0.6). The difference was significant, *t*(34) = −6.498, *p* < .001, and the effect size was large (Cohen's *d* = 1.352). Individual item means for these respondents at pre- and posttest are displayed in Table 2 with internal consistency reliability coefficients. On the pretest, learners showed the highest confidence at recognizing atypical genitalia in a newborn. This was the item that showed the smallest increase in confidence. Increases in confidence were greatest around the initial approach to evaluating suspected DSD in a newborn and how to provide initial counseling to the family of a newborn with atypical genitalia or suspected DSD using principles of shared decision-making. Learners were enthusiastic about wanting to see the activity repeated, with an average reaction of 7.9 (*SD* = 2.3) on a scale of 0 to 10, where 10 was a very strong yes (*Absolutely!*).

**Table 2.**
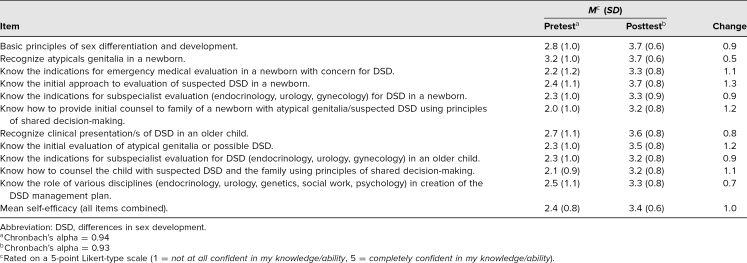
Mean Response for Each Self-Efficacy Item and Scale Reliability for Participants who Completed Both Measures (*n* = 35)

### Qualitative

To further explore student perspectives of the activity, we inductively coded^11^ the open-ended short-answer responses (*n* = 42) from the evaluation survey. Three key themes emerged from this analysis of learners’ biggest takeaway and suggestions for additional content.

#### Learning about DSD-specific concepts was a key outcome

Learners highlighted that the activity assisted them in gaining a better understanding of DSD and/or learning about new concepts related to DSD. For example, one student stated “Better understanding etiologies, manifestations, and clinical approaches.” This suggested that the TBL activity was an effective pedagogical tool in teaching pediatric residents about DSD. Additionally, it reaffirmed the results of the evaluation measure where the majority (80%) of learners indicated that the pilot curriculum was an appropriate tool for improving DSD skills and knowledge.

#### Learners found a review of general medical concepts to be a valuable component of the activity

Although a majority of student responses addressed learning new concepts specific to DSD, the second theme that emerged illustrated that learners also placed value in reviewing relevant general medical concepts. Example comments included: “Good review of hormonal pathways,” and, “Refresher on adrenal and differentiation of pathways.” This suggested that a key component for teaching learners about DSD-specific content was also connecting these topics and reinforcing general medical knowledge.

#### The opportunity to discuss complex and challenging concepts was a valuable component of the activity

While less prominent than the first two constructs, the emergence of this theme suggested that some learners appreciated engaging with the complex topics that were integrated into the activity. Example comments included: “Lots of active discussion/controversy re. psychosocial aspects of DSD management,” and, “Controversy in how to approach surgical decision-making.” This suggested that learners recognized the complexity of caring for patients with DSD, a finding that was also reflected in the significant change observed in self-confidence about counseling, using shared decision-making, and in their knowledge about the role of other disciplines in management of DSD. For instance, during the group application activity, for case 1 (first question) groups chose between option 3 (delay adolescence) and choice 4 (surgery during infancy) which led to a discussion about current controversies around timing of surgery. For case 2, there was variation in team responses around expected gonadotropin hormone results in a patient with androgen insensitivity which led to a debate and discussion with final agreement for the preferred response. Further, team responses differed for the third question of case 2 and this led to an animated discussion on ethics of not disclosing the diagnosis to an adolescent as well as expression of interest in learning about shared decision-making.

## Discussion

We found that TBL was a valuable educational strategy to teach pediatric residents and medical students about care of patients with DSD. Both pediatric residents and medical students who participated reported minimal prior training about DSD, confirming that the exercise met a need. Low iRAT and gRAT scores also underscored knowledge gaps. For instance, in question 3, the majority of learners were distracted by medical conditions in the nearest family member and seemed to find it challenging to recognize that history of a relative might be relevant. Similarly, in question 4, the majority of learners did not seem to know the importance of karyotype amongst available choices since they picked other incorrect responses for initial testing. Both groups demonstrated improved knowledge and increased comfort in managing patients with DSD following the full activity, suggesting that the exercise filled the educational gap. The timeliness of this activity cannot be overemphasized given the recent controversies around management of DSD which can accentuate gaps in care for an individual with DSD, from lack of knowledge of foundational concepts to lack of familiarity with principles of management of DSD.

In addition, from the perspective of inclusivity, it is important to highlight that medical practitioners must be very cognizant of concerns of individuals with DSD and the disparities they face, empowering the individual and keeping their benefit in mind is essential. An equally important point of learning from the authors’ work caring for this population has been that cultural beliefs of a family play a huge role in this as much as any other chronic illness and respecting and recognizing those beliefs goes a long way in building trust with the family for ultimate benefit of the child. It is important to make sure that families of children with DSD are not left out of the dialogue (irrespective of their views on DSD) and/or feel marginalized to the point of leaving professional medical care and seeking help elsewhere.

TBL activities engage learners at various levels of training. Working as a team, pediatric residents and medical students were more successful at answering questions about the learning material than they were individually. They valued gaining a better understanding of concepts through the opportunity to discuss practical challenges in patient care via group application activities as discussed in the results above around approach to the patient, expected hormone levels, and ethics of delaying disclosure of the diagnosis to an adolescent. They were required to actively engage in critical thinking and discussion. This gave medical students and early residents an opportunity to share their recently acquired knowledge about basic anatomy and physiology, while more senior residents were able to share and apply their experience with patient care. This mutual exchange likely contributed to the overall increase in confidence around managing patients with DSD. Residents appeared most engaged in the discussion on clinical approach and determining etiology of a DSD, as well as the practical challenges of management of DSD as evidenced by qualitative responses. The activity may be improved by increasing the time spent on activities to enhance shared decision-making, particularly around dilemmas a patient/family faces when contending with DSD.

To our knowledge, this is the only educational activity designed for residents as learners and topic of DSD in the *MedEdPORTAL* curriculum collection. We found one problem-based learning activity for medical students that used a case of complete androgen sensitivity^12^ and another that discussed congenital adrenal hyperplasia (CAH) as part of a medical student physiology lecture series.^13^ Another curriculum for emergency medicine residents included a case of CAH as part of their focus on teaching immediate resuscitation skills.^14^ Given the recent controversies and changing paradigms around DSD management, the activity's inclusion of both the knowledge component for evaluation/management as well as exploration of principles of decision making in DSD management using a TBL approach was innovative. Also, this makes it uniquely positioned as a beneficial curriculum for this complex topic to both medical students and residents.

We acknowledge some important limitations. First, given this didactic is part of a 3-year rotating curriculum, we were able to pilot this only once with our cohort of 36 learners. Therefore, we were not able to make specific inferences about learner levels or differences between residents and medical students. Further, we did not perform a detailed diagnostic analysis to discern reasons for incorrect responses on the RAT given the time limitations for the activity. We speculate these may be related to gaps in knowledge around DSD as reflected in the qualitative analysis of learner's reflections in results. Second, the self-efficacy measure we used for evaluation purposes was one of our own creation. While our measures of reliability were high for the pretest and posttest responses, the tool has not yet been validated in other samples. Another limitation was the relatively low scores on iRAT and gRAT, specifically with questions around history, physical exam, and initial testing, which suggested a lack of prereading by most learners prior to the didactic. This is a common challenge in clinical learners, and we anticipated it due to the added complexity and cognitive difficulty inherent to DSD concepts; thus, we chose publications that we thought would be easy reads from a resident learner's perspective. We will likely change the required reading to a single alternate article^1^ in our next iteration of the activity. Programs that have options for external rewards such as gift cards in small denominations or dedicated time for prereading may choose to use those resources to incentivize preclass preparation and signify the importance of the topic. We believe that activities such as this TBL provide the opportunity for learners to have a successful learning outcome for difficult topics such as DSD even with minimal prior preparation. Due to the nature of the project, assessing long-term knowledge retention was outside the scope of this study. This module would benefit from future studies in knowledge retention compared to a traditional didactic education session.

With respect to the activity, one of the primary goals of TBL is to facilitate team cohesion and foster successful problem solving through guided activities.^5^ TBL typically relies on repeated encounters for learner groups to form into ideal, high-performance teams.^7^ Unfortunately, this amount of contact time is rarely available in residency curricula and was not available for the purpose of this module. We did, however, observe that team members were generally well engaged, with appropriate individual participation in discussion. We believe this time barrier was minimized because most learners had already had an opportunity to interact with each other in the clinical environment prior to the activity. In fact, this activity can be modified to fit a typical 60-minute lecture slot for any program considering including it in their residency curriculum by decreasing time in individual readiness assessment from 25 to 12 minutes and eliminating the pre- and postactivity assessments. Further studies directed at evaluating the effectiveness of TBL in low-contact time settings would be helpful.

In summary, this TBL activity was designed to provide instruction in the management of DSD. The module engaged learners in individual assessment followed by small-group application exercises, and participants expressed improved knowledge and comfort in management of DSD in children. Evidence we collected supported this as a valuable educational strategy to enhance knowledge of DSD care for learners at various levels.

## Appendices

Team Materials List.docxPre-Post Assessment iRAT Response Form.docxTBL Activity Slides.pptxStudent RAT.docxFacilitator RAT.docxFacilitator Team Application Activity.docxStudent Team Application Activity.docxAdrenal Enzyme Pathway Diagram.docxPrader Scale Handout.docx
All appendices are peer reviewed as integral parts of the Original Publication.
