# Supplementary material for: Increasing Knowledge and Self-Efficacy on Differences in Sex Development (DSD): A Team-Based Learning Activity for Pediatric Residents
Source: MedEdPORTAL. 2021 Feb 23;17:11105. doi: 10.15766/mep_2374-8265.11105 (PMC7901252; doi:10.15766/mep_2374-8265.11105)
Supplement: Supplementary file 1 — Team Materials List.docxPre-Post Assessment iRAT Response Form.docxTBL Activity Slides.pptxStudent RAT.docxFacilitator RAT.docxFacilitator Team Application Activity.docxStudent Team Application Activity.docxAdrenal Enzyme Pathway Diagram.docxPrader Scale Handout.docx [file mep_2374-8265.11105-s001.zip › A. Team Materials List.docx]

Differences in Sex Development (DSD):
Team-Based Learning Module for Pediatric Residents

**Materials to Include in Each Team’s Folder for the TBL**

1. Stapled worksheet series (1 for each learner): pretest survey on page 1, individual RAT response sheet on page 2, post-activity assessment on the reverse side of page 2 [Appendix B]
2. Readiness Assurance Test – Student Version (1 for each learner) [Appendix D]
3. IF/AT card (1 for the team)
4. Team Application Activity – Student Version (1 for each learner) [Appendix G]
5. Adrenal enzyme pathway handout (1 for each learner) [Appendix H]
6. Prader scale handout (1 for each learner) [Appendix I]
