# Supplementary material for: Increasing Knowledge and Self-Efficacy on Differences in Sex Development (DSD): A Team-Based Learning Activity for Pediatric Residents
Source: MedEdPORTAL. 2021 Feb 23;17:11105. doi: 10.15766/mep_2374-8265.11105 (PMC7901252; doi:10.15766/mep_2374-8265.11105)
Supplement: Supplementary file 1 — Team Materials List.docxPre-Post Assessment iRAT Response Form.docxTBL Activity Slides.pptxStudent RAT.docxFacilitator RAT.docxFacilitator Team Application Activity.docxStudent Team Application Activity.docxAdrenal Enzyme Pathway Diagram.docxPrader Scale Handout.docx [file mep_2374-8265.11105-s001.zip › B. Pre-Post Assessment iRAT Response Form.docx]

Group Name/Number

Differences in Sex Development (DSD):
Team-Based Learning Module for Pediatric Residents

**Pre-activity Self-Assessment**

Please respond to the items below. There are no right or wrong answers. Your responses will be used to assess and revise curriculum development around knowledge, skills, or attitudes of learners toward DSD.

| Rate your level of confidence in your own  knowledge, understanding, or ability on  each of the following aspects of DSD management. | **NOT AT ALL CONFIDENT in my knowledge /ability** |  |  |  | **COMPLETELY CONFIDENT in my knowledge /ability** |
| --- | --- | --- | --- | --- | --- |
|  | **1** | **2** | **3** | **4** | **5** |
| 1. Basic principles of sex differentiation and development | 🔾 | 🔾 | 🔾 | 🔾 | 🔾 |
| 1. Recognize atypical genitalia in a newborn | 🔾 | 🔾 | 🔾 | 🔾 | 🔾 |
| 1. Know the indications for emergency medical evaluation in a newborn with concern for DSD | 🔾 | 🔾 | 🔾 | 🔾 | 🔾 |
| 1. Know the initial approach to evaluation of suspected DSD in a newborn | 🔾 | 🔾 | 🔾 | 🔾 | 🔾 |
| 1. Know the indications for subspecialist evaluation (Endocrinology, Urology, Gynecology) for DSD in a newborn | 🔾 | 🔾 | 🔾 | 🔾 | 🔾 |
| 1. Know how to provide initial counsel to family of a newborn with atypical genitalia/suspected DSD using principles of shared decision making | 🔾 | 🔾 | 🔾 | 🔾 | 🔾 |
| 1. Recognize clinical presentation/s of variation in sex development in an older child | 🔾 | 🔾 | 🔾 | 🔾 | 🔾 |
| 1. Know the initial evaluation of atypical genitalia or possible DSD | 🔾 | 🔾 | 🔾 | 🔾 | 🔾 |
| 1. Know the indications for subspecialist evaluation for DSD (Endocrinology, Urology, Gynecology) in an older child | 🔾 | 🔾 | 🔾 | 🔾 | 🔾 |
| 1. Know how to counsel the child with suspected DSD and the family using principles of shared decision making | 🔾 | 🔾 | 🔾 | 🔾 | 🔾 |
| 1. Know the role of various disciplines (endocrinology, urology, genetics, social work, psychology) in creation of the DSD management plan | 🔾 | 🔾 | 🔾 | 🔾 | 🔾 |

Please select your year of training:

| 🔾 Medical Student | 🔾 PGY-1 | 🔾 PGY-2 | 🔾 PGY-3 | 🔾 PGY-4 |
| --- | --- | --- | --- | --- |

Have you received DSD education for a personal reason (self or immediate family member)?

| 🔾 Yes | 🔾 No |
| --- | --- |

How many hours of didactic education on DSDs did you receive or have you received during medical school?

| 🔾 1 hour | 🔾 2-4 hours | 🔾 more than 4 hours |
| --- | --- | --- |

How many patients have you seen with differences of sex development?

| 🔾 0 | 🔾 1 | 🔾 2-4 | 🔾 more than 5 |
| --- | --- | --- | --- |

[Intentionally left blank]

**Individual Readiness Assurance Test**

**Response Worksheet**

Enter the letter corresponding to your response for each question.


**Post-activity Self-Assessment and Evaluation**

Please respond to the items below. There are no right or wrong answers. Your responses will be used to assess and revise curriculum development around knowledge, skills, or attitudes of learners toward DSD.

| Rate your level of confidence in your own  knowledge, understanding, or ability on  each of the following aspects of DSD management. | **NOT AT ALL CONFIDENT in my knowledge /ability** |  |  |  | **COMPLETELY CONFIDENT in my knowledge /ability** |
| --- | --- | --- | --- | --- | --- |
|  | **1** | **2** | **3** | **4** | **5** |
| 1. Basic principles of sex differentiation and development | 🔾 | 🔾 | 🔾 | 🔾 | 🔾 |
| 1. Recognize atypical genitalia in a newborn | 🔾 | 🔾 | 🔾 | 🔾 | 🔾 |
| 1. Know the indications for emergency medical evaluation in a newborn with concern for DSD | 🔾 | 🔾 | 🔾 | 🔾 | 🔾 |
| 1. Know the initial approach to evaluation of suspected DSD in a newborn | 🔾 | 🔾 | 🔾 | 🔾 | 🔾 |
| 1. Know the indications for subspecialist evaluation (Endocrinology, Urology, Gynecology) for DSD in a newborn | 🔾 | 🔾 | 🔾 | 🔾 | 🔾 |
| 1. Know how to provide initial counsel to family of a newborn with atypical genitalia/suspected DSD using principles of shared decision making | 🔾 | 🔾 | 🔾 | 🔾 | 🔾 |
| 1. Recognize clinical presentation/s of variation in sex development in an older child | 🔾 | 🔾 | 🔾 | 🔾 | 🔾 |
| 1. Know the initial evaluation of atypical genitalia or possible DSD | 🔾 | 🔾 | 🔾 | 🔾 | 🔾 |
| 1. Know the indications for subspecialist evaluation for DSD (Endocrinology, Urology, Gynecology) in an older child | 🔾 | 🔾 | 🔾 | 🔾 | 🔾 |
| 1. Know how to counsel the child with suspected DSD and the family using principles of shared decision making | 🔾 | 🔾 | 🔾 | 🔾 | 🔾 |
| 1. Know the role of various disciplines (endocrinology, urology, genetics, social work, psychology) in creation of the DSD management plan | 🔾 | 🔾 | 🔾 | 🔾 | 🔾 |

What was your biggest takeaway from this activity?

Would you like to repeat this activity next year? Scale = 0 (no way!) to 10 (absolutely!)

What would you like to see added to this activity given the time allotted?
