# Supplementary material for: Increasing Knowledge and Self-Efficacy on Differences in Sex Development (DSD): A Team-Based Learning Activity for Pediatric Residents
Source: MedEdPORTAL. 2021 Feb 23;17:11105. doi: 10.15766/mep_2374-8265.11105 (PMC7901252; doi:10.15766/mep_2374-8265.11105)
Supplement: Supplementary file 1 — Team Materials List.docxPre-Post Assessment iRAT Response Form.docxTBL Activity Slides.pptxStudent RAT.docxFacilitator RAT.docxFacilitator Team Application Activity.docxStudent Team Application Activity.docxAdrenal Enzyme Pathway Diagram.docxPrader Scale Handout.docx [file mep_2374-8265.11105-s001.zip › C. TBL Activity Slides.pptx]

## Slide 1
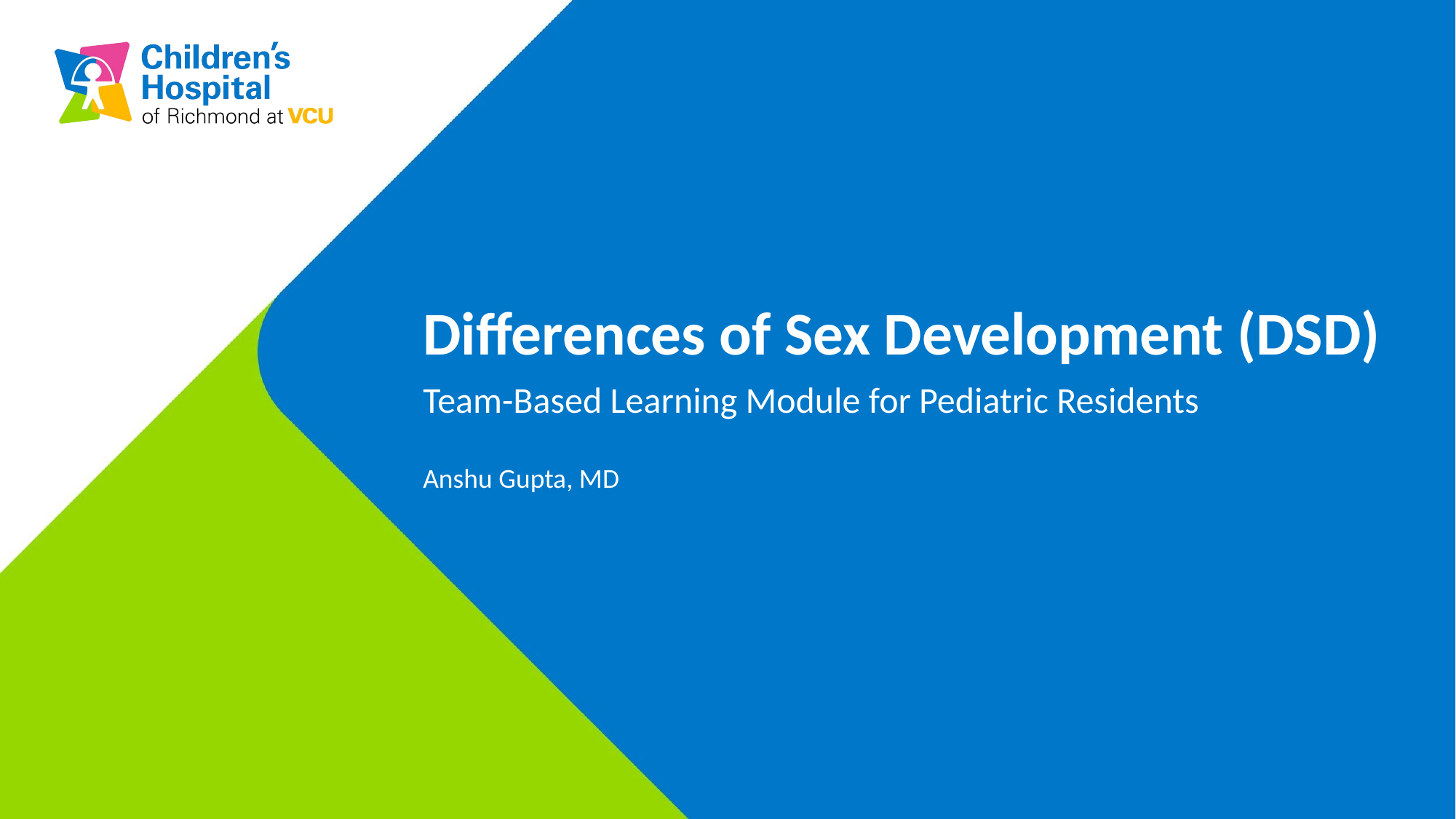

# Differences of Sex Development (DSD)
Team-Based Learning Module for Pediatric Residents
Anshu Gupta, MD

## Slide 2
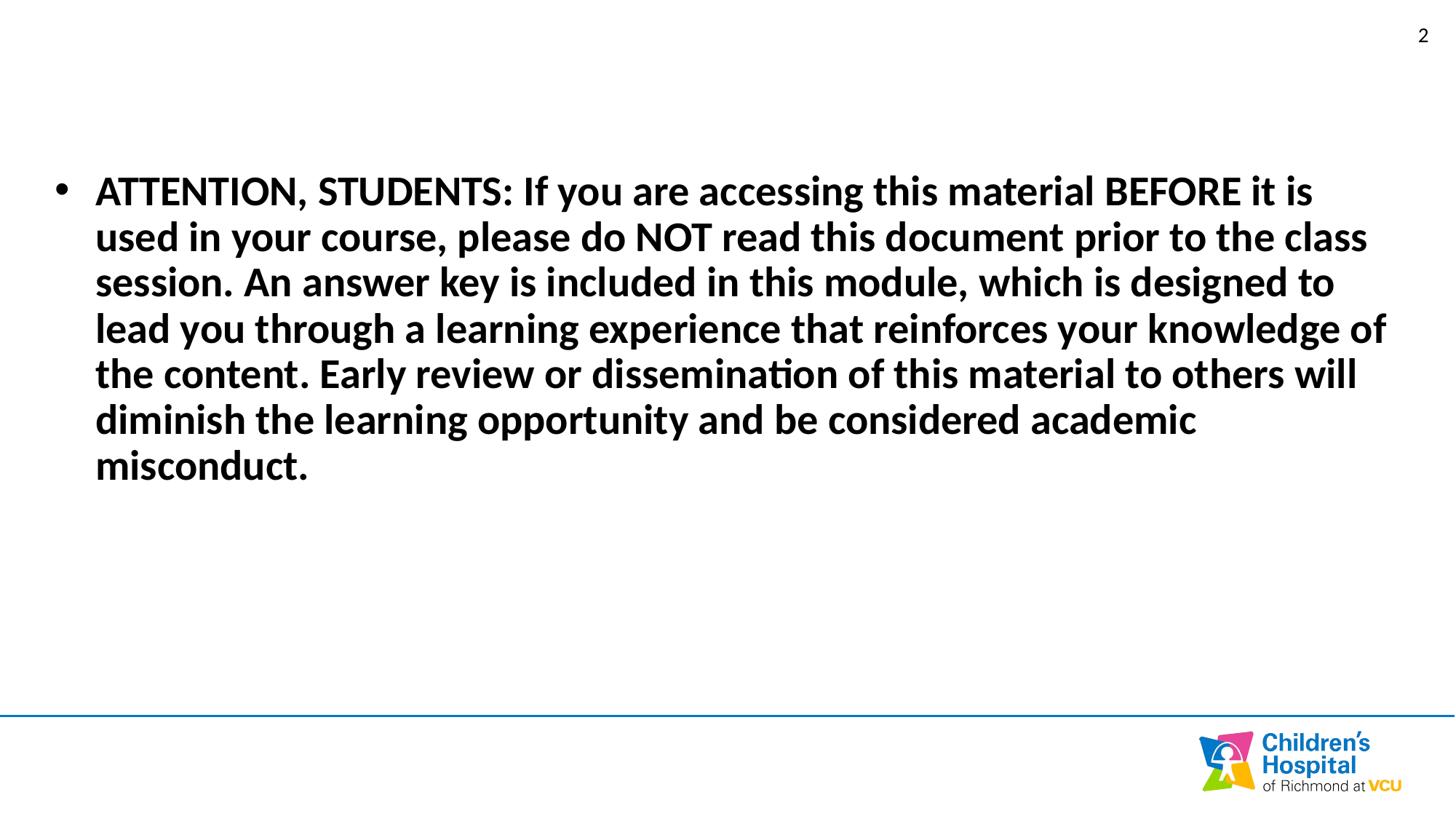

2
ATTENTION, STUDENTS: If you are accessing this material BEFORE it is used in your course, please do NOT read this document prior to the class session. An answer key is included in this module, which is designed to lead you through a learning experience that reinforces your knowledge of the content. Early review or dissemination of this material to others will diminish the learning opportunity and be considered academic misconduct.

## Slide 3
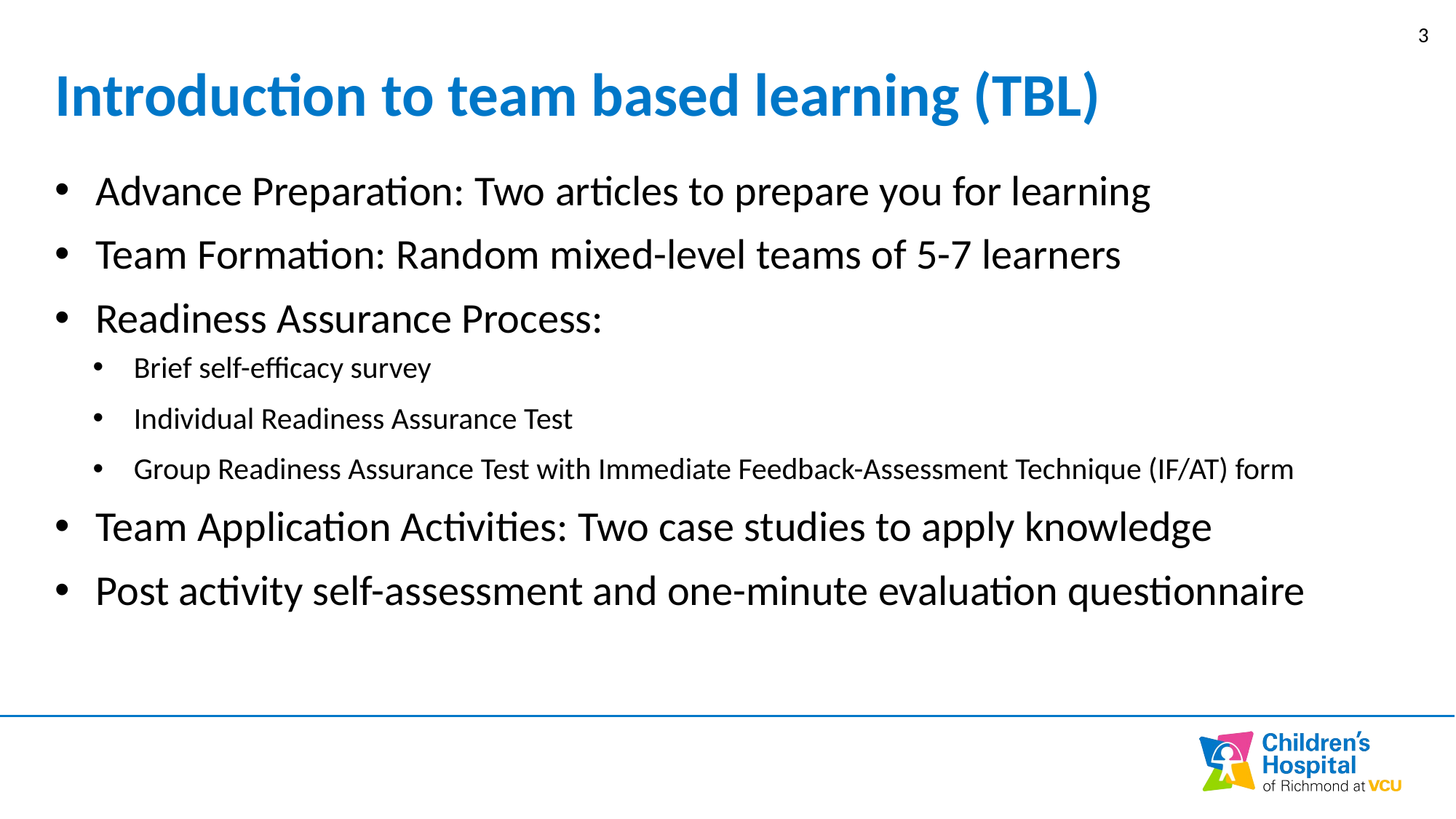

3
# Introduction to team based learning (TBL)
Advance Preparation: Two articles to prepare you for learning
Team Formation: Random mixed-level teams of 5-7 learners
Readiness Assurance Process:
Brief self-efficacy survey
Individual Readiness Assurance Test
Group Readiness Assurance Test with Immediate Feedback-Assessment Technique (IF/AT) form
Team Application Activities: Two case studies to apply knowledge
Post activity self-assessment and one-minute evaluation questionnaire

## Slide 4
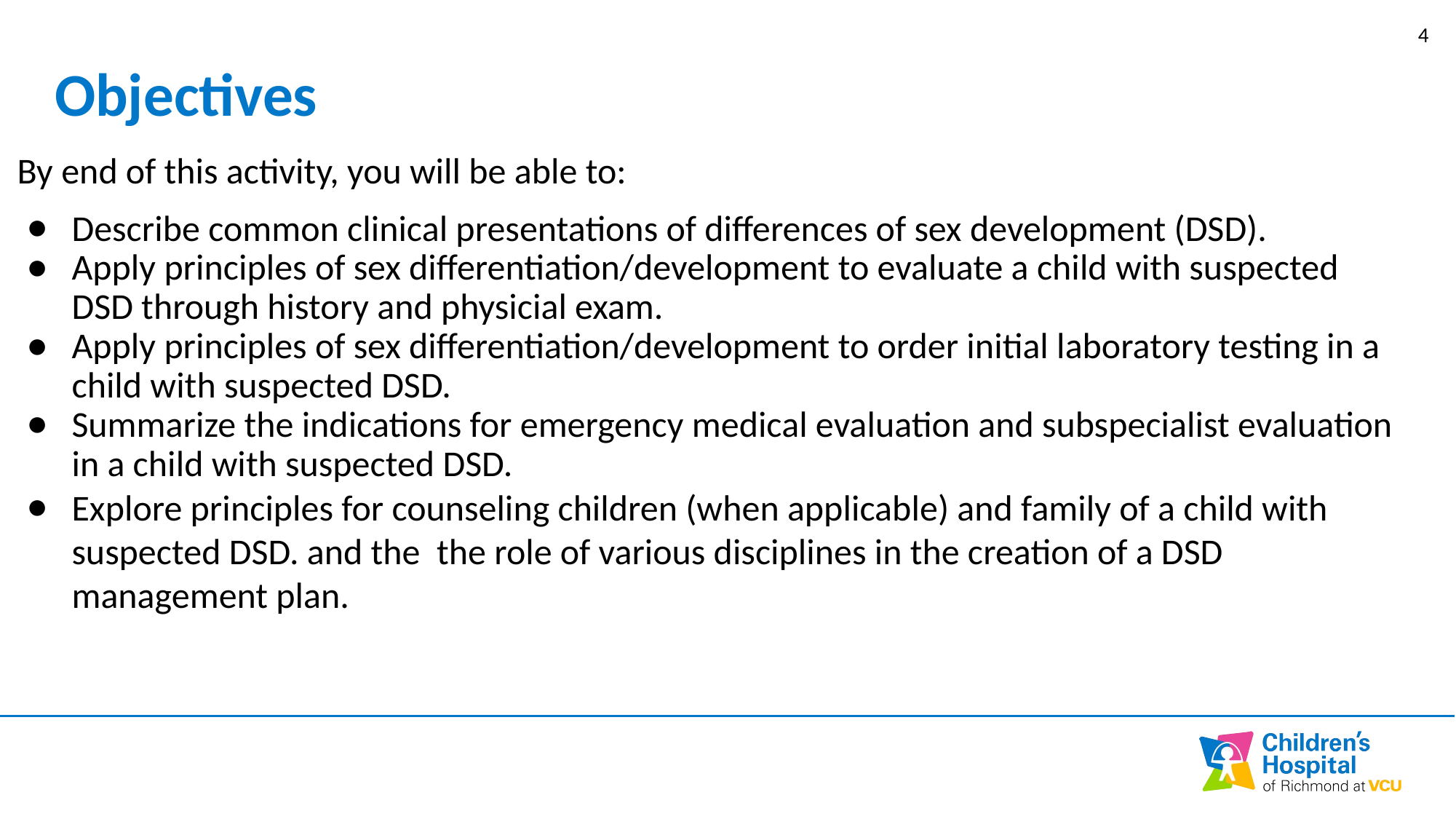

4
# Objectives
By end of this activity, you will be able to:
Describe common clinical presentations of differences of sex development (DSD).
Apply principles of sex differentiation/development to evaluate a child with suspected DSD through history and physicial exam.
Apply principles of sex differentiation/development to order initial laboratory testing in a child with suspected DSD.
Summarize the indications for emergency medical evaluation and subspecialist evaluation in a child with suspected DSD.
Explore principles for counseling children (when applicable) and family of a child with suspected DSD. and the the role of various disciplines in the creation of a DSD management plan.

## Slide 5
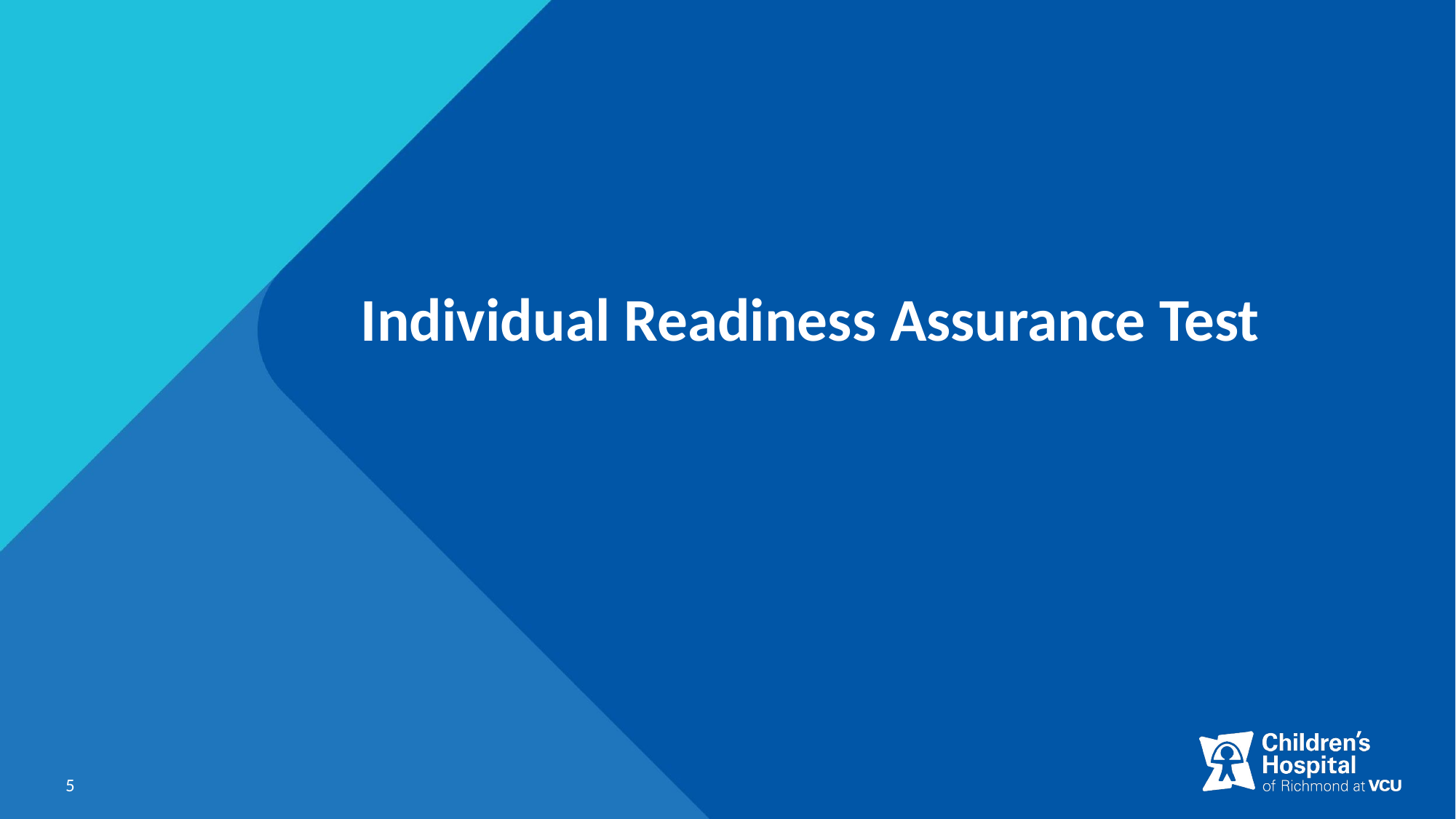

# Individual Readiness Assurance Test
5

## Slide 6
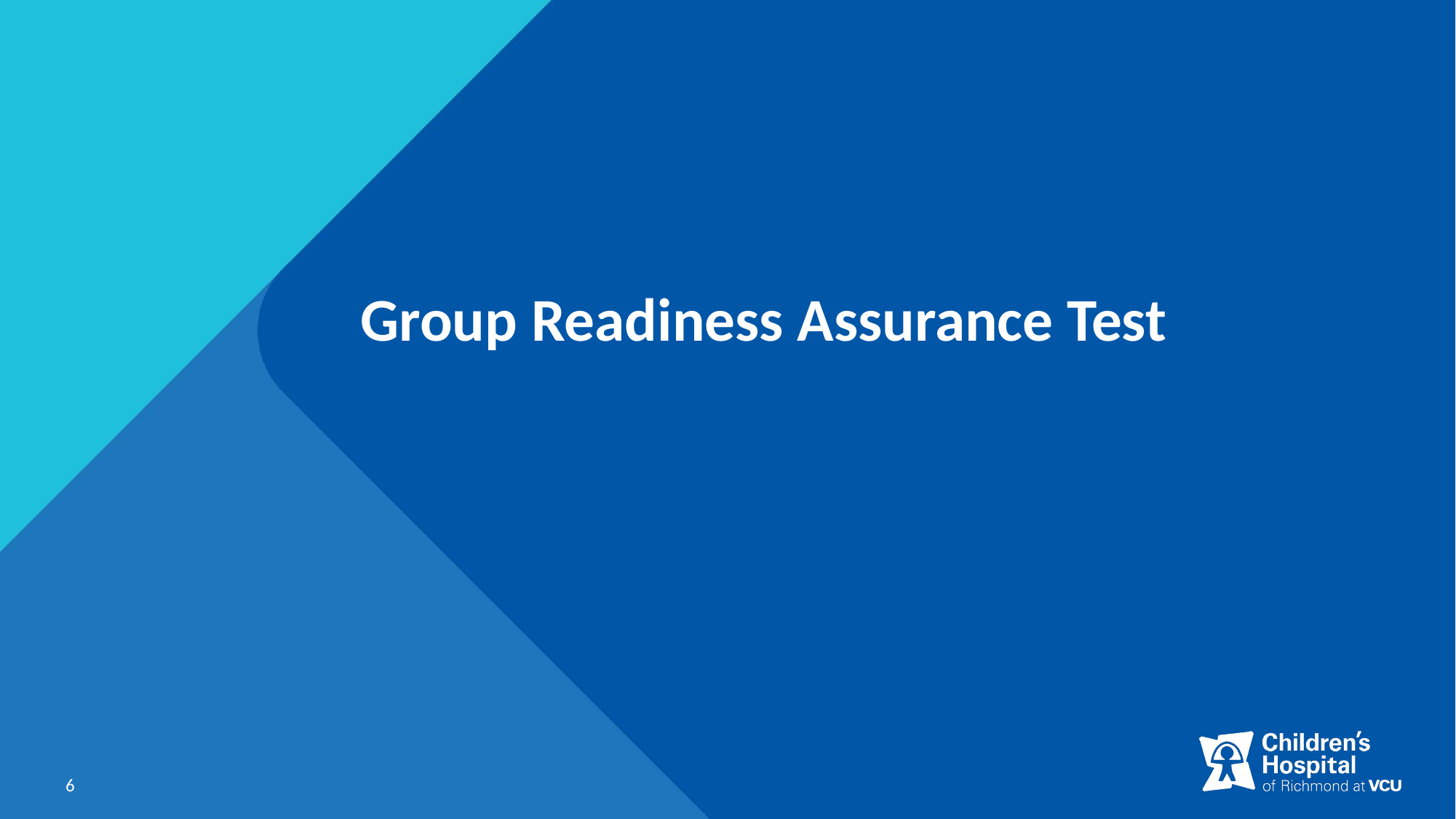

# Group Readiness Assurance Test
6

## Slide 7
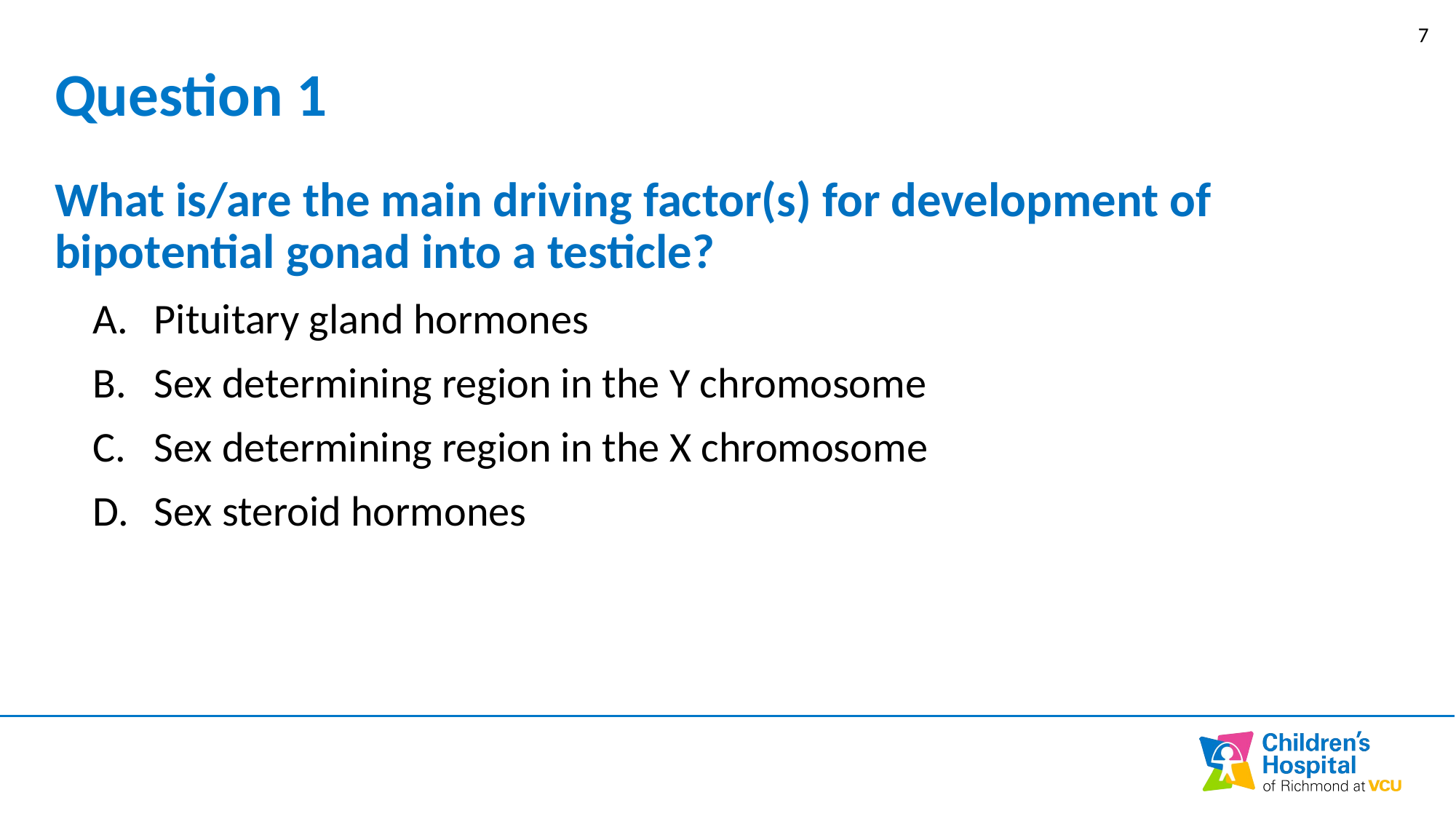

7
# Question 1
What is/are the main driving factor(s) for development of bipotential gonad into a testicle?
Pituitary gland hormones
Sex determining region in the Y chromosome
Sex determining region in the X chromosome
Sex steroid hormones

## Slide 8
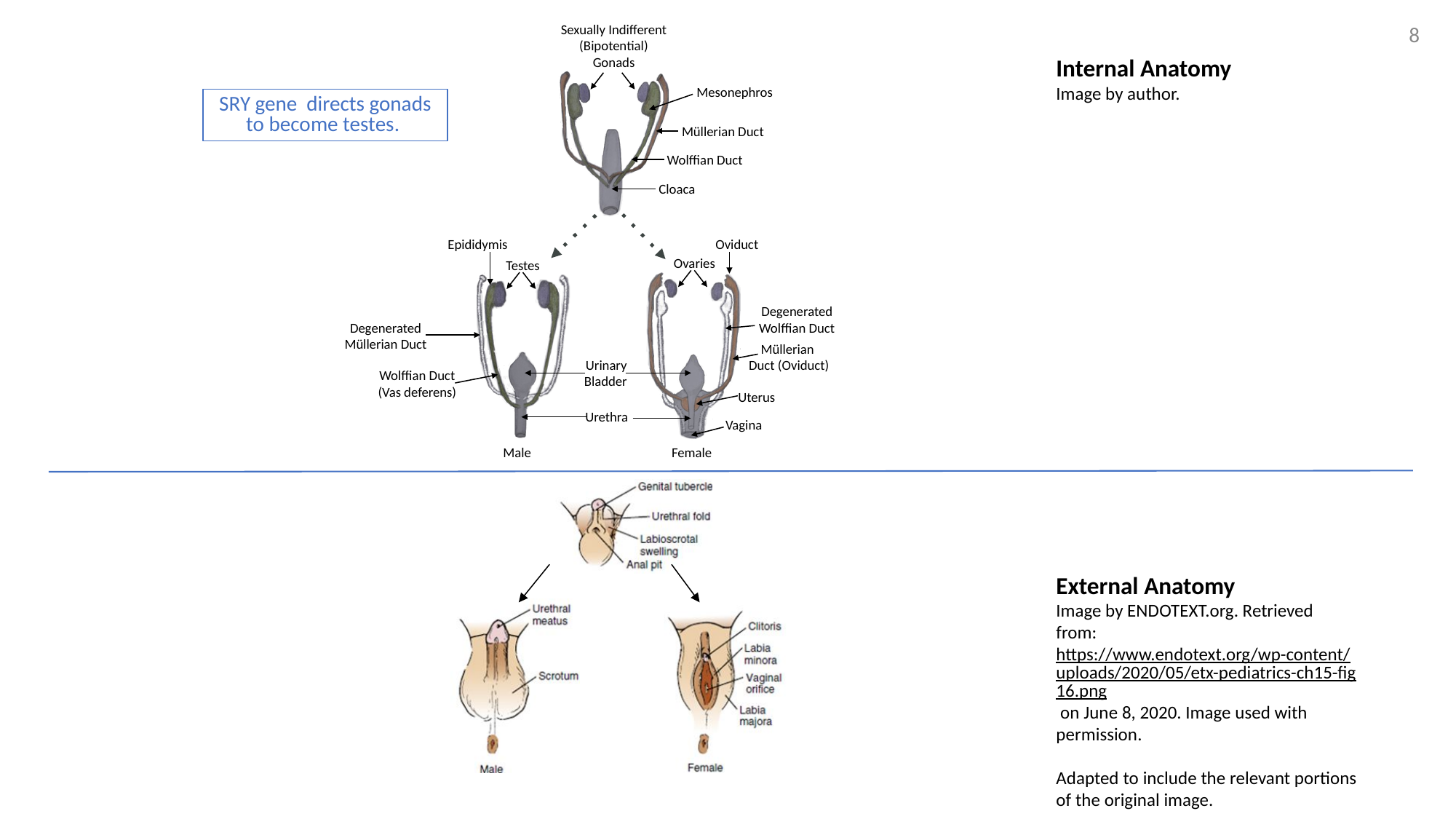

8
Sexually Indifferent
(Bipotential)
Gonads
Mesonephros
Müllerian Duct
Wolffian Duct
Cloaca
Epididymis
Oviduct
Ovaries
Testes
Degenerated Wolffian Duct
Degenerated Müllerian Duct
Müllerian
Duct (Oviduct)
Urinary Bladder
Wolffian Duct (Vas deferens)
Uterus
Urethra
Vagina
Male
Female
Internal Anatomy
Image by author.
SRY gene directs gonads to become testes.
External Anatomy
Image by ENDOTEXT.org. Retrieved from: https://www.endotext.org/wp-content/uploads/2020/05/etx-pediatrics-ch15-fig16.png on June 8, 2020. Image used with permission.
Adapted to include the relevant portions of the original image.

## Slide 9
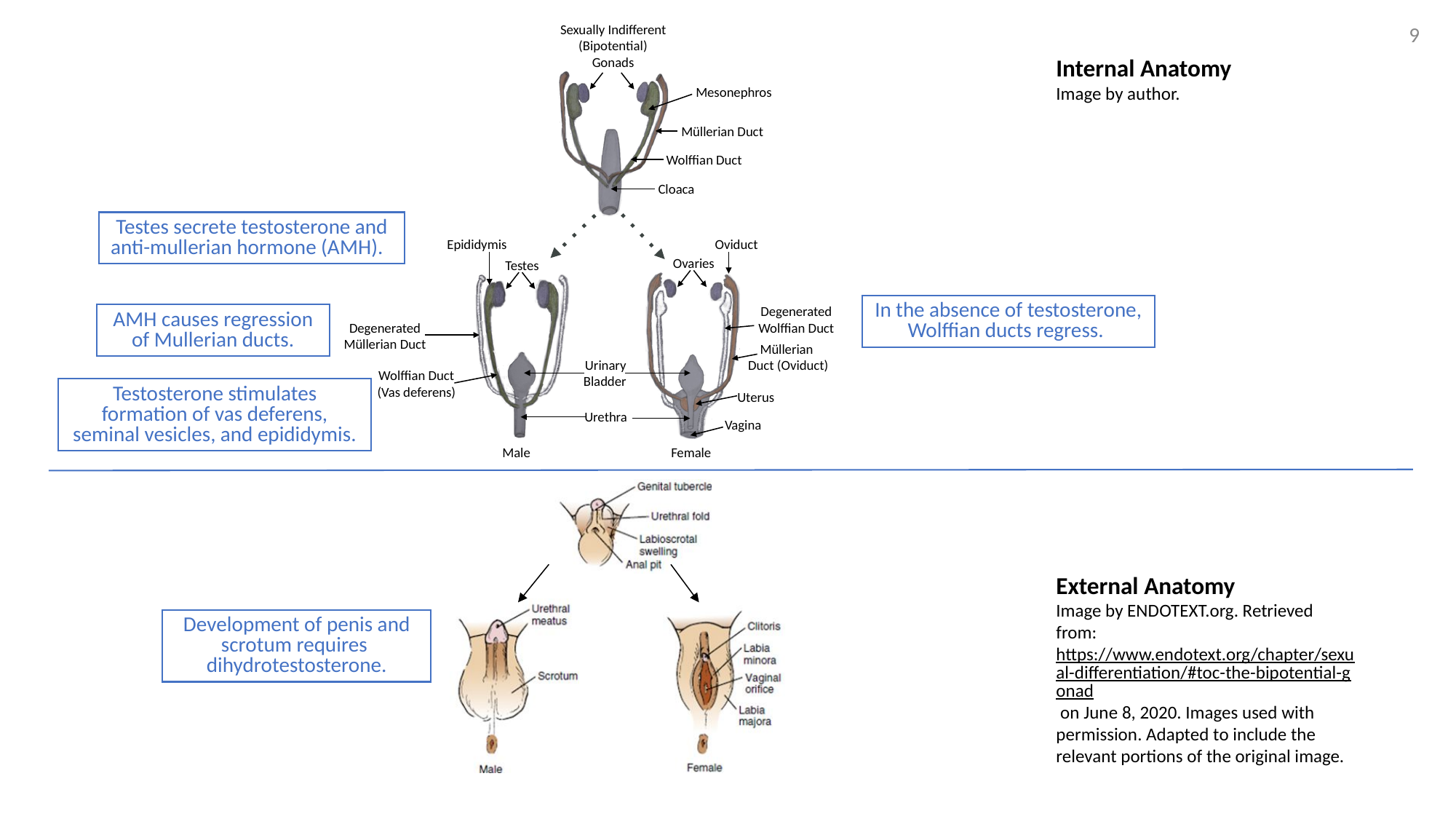

9
Sexually Indifferent
(Bipotential)
Gonads
Mesonephros
Müllerian Duct
Wolffian Duct
Cloaca
Epididymis
Oviduct
Ovaries
Testes
Degenerated Wolffian Duct
Degenerated Müllerian Duct
Müllerian
Duct (Oviduct)
Urinary Bladder
Wolffian Duct (Vas deferens)
Uterus
Urethra
Vagina
Male
Female
Internal Anatomy
Image by author.
Testes secrete testosterone and anti-mullerian hormone (AMH).
In the absence of testosterone, Wolffian ducts regress.
AMH causes regression of Mullerian ducts.
Testosterone stimulates formation of vas deferens, seminal vesicles, and epididymis.
External Anatomy
Image by ENDOTEXT.org. Retrieved from: https://www.endotext.org/chapter/sexual-differentiation/#toc-the-bipotential-gonad on June 8, 2020. Images used with permission. Adapted to include the relevant portions of the original image.
Development of penis and scrotum requires
dihydrotestosterone.

## Slide 10
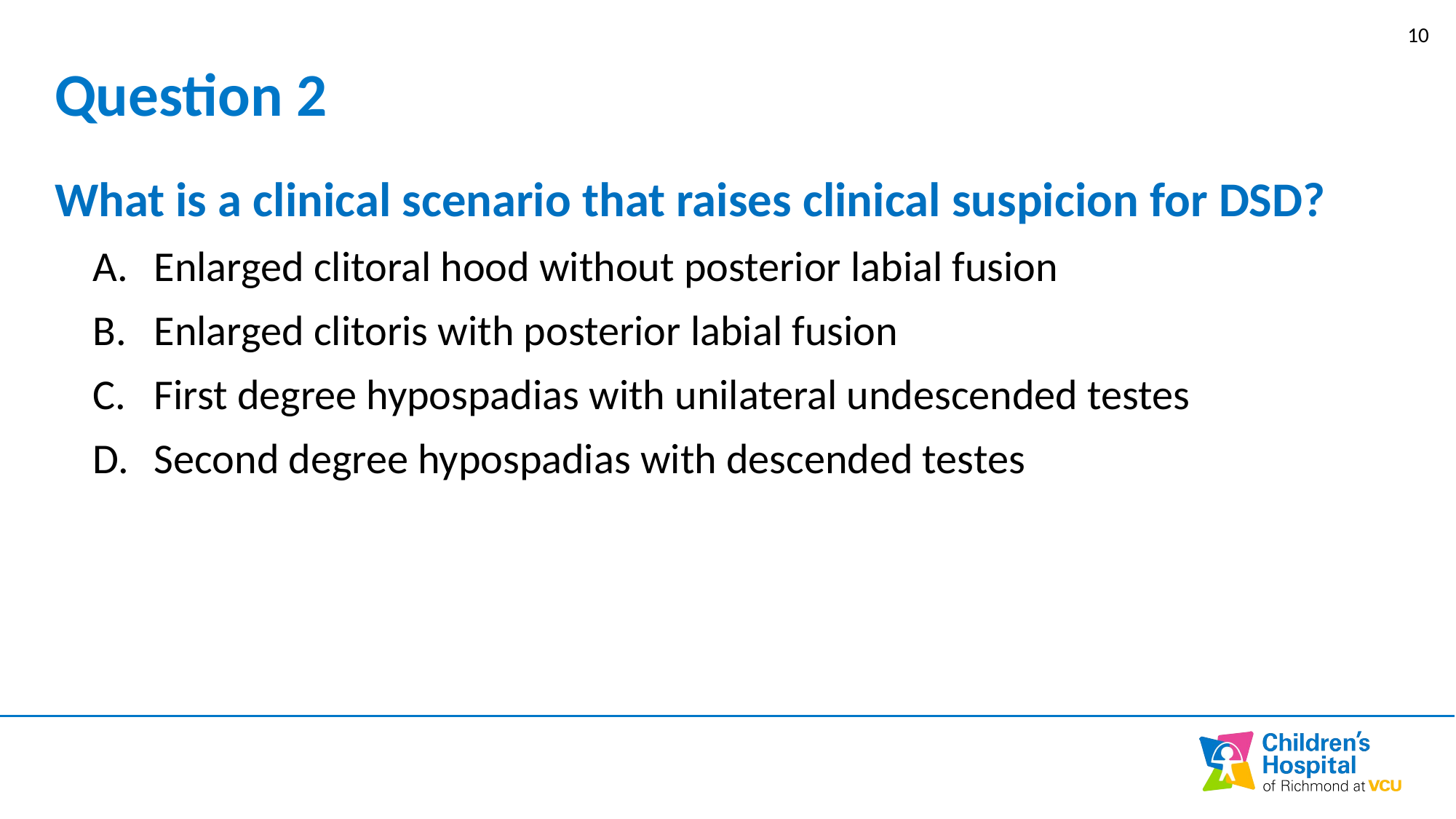

10
# Question 2
What is a clinical scenario that raises clinical suspicion for DSD?
Enlarged clitoral hood without posterior labial fusion
Enlarged clitoris with posterior labial fusion
First degree hypospadias with unilateral undescended testes
Second degree hypospadias with descended testes

## Slide 11
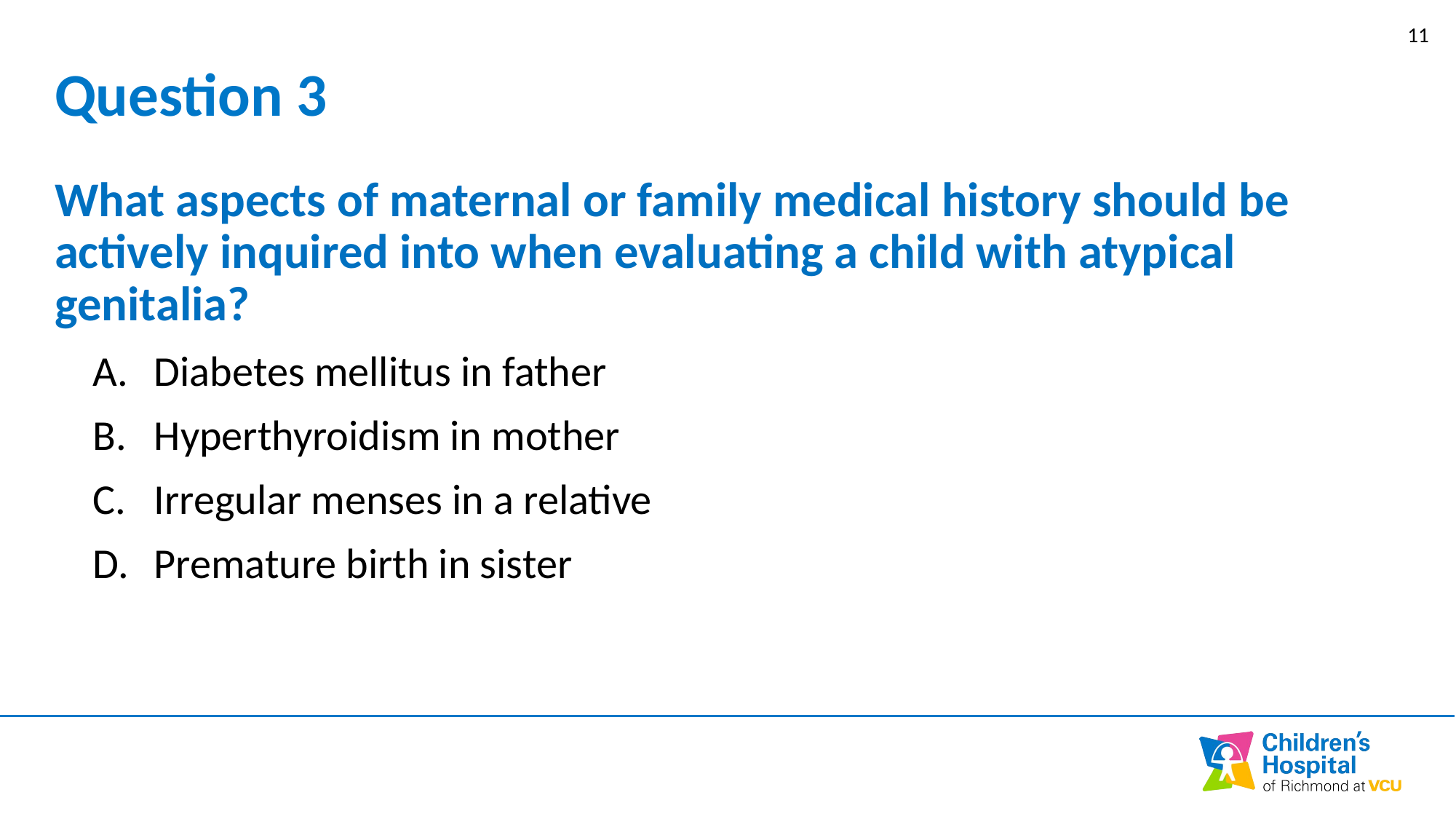

11
# Question 3
What aspects of maternal or family medical history should be actively inquired into when evaluating a child with atypical genitalia?
Diabetes mellitus in father
Hyperthyroidism in mother
Irregular menses in a relative
Premature birth in sister

## Slide 12
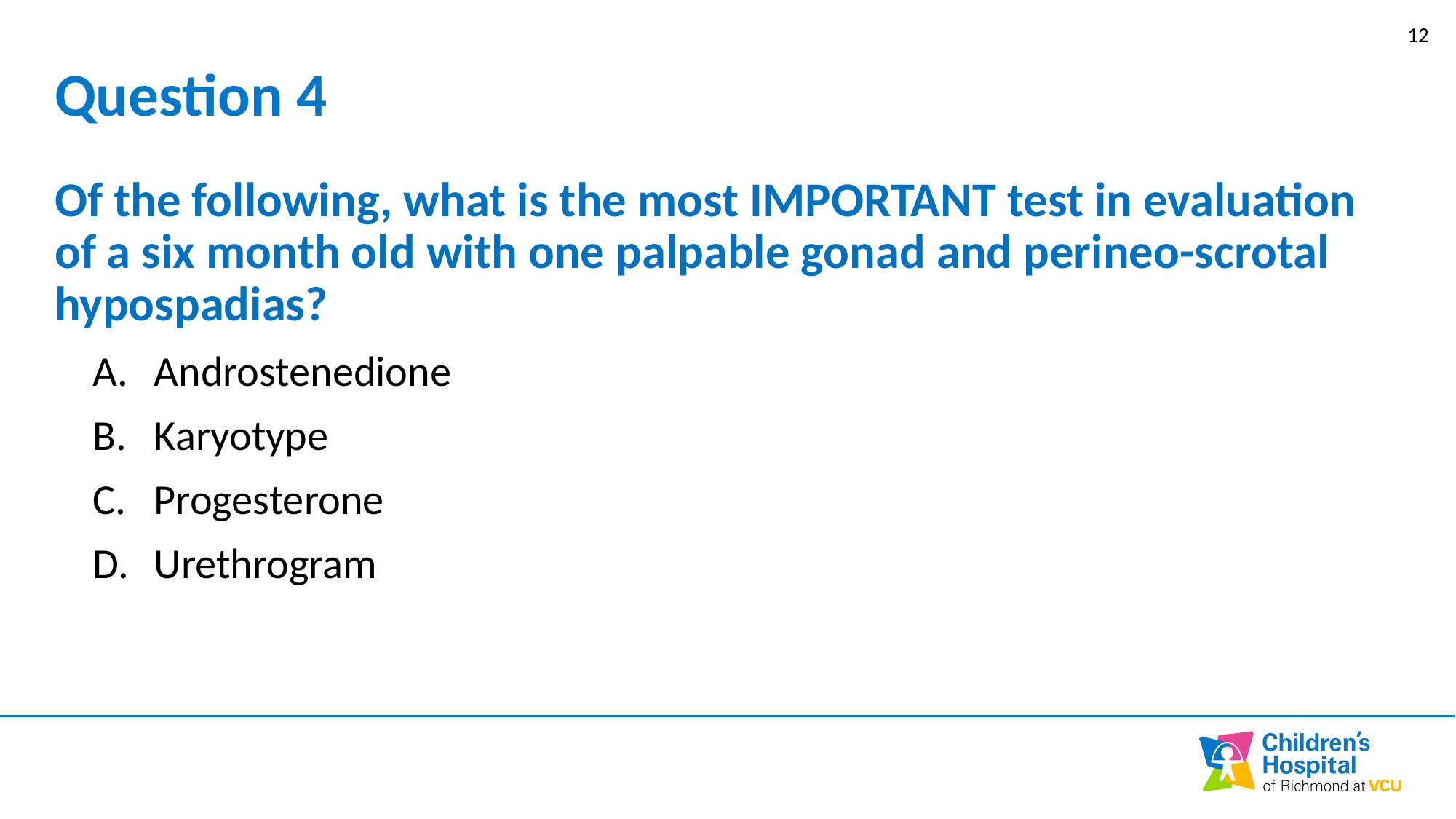

12
# Question 4
Of the following, what is the most IMPORTANT test in evaluation of a six month old with one palpable gonad and perineo-scrotal hypospadias?
Androstenedione
Karyotype
Progesterone
Urethrogram

## Slide 13
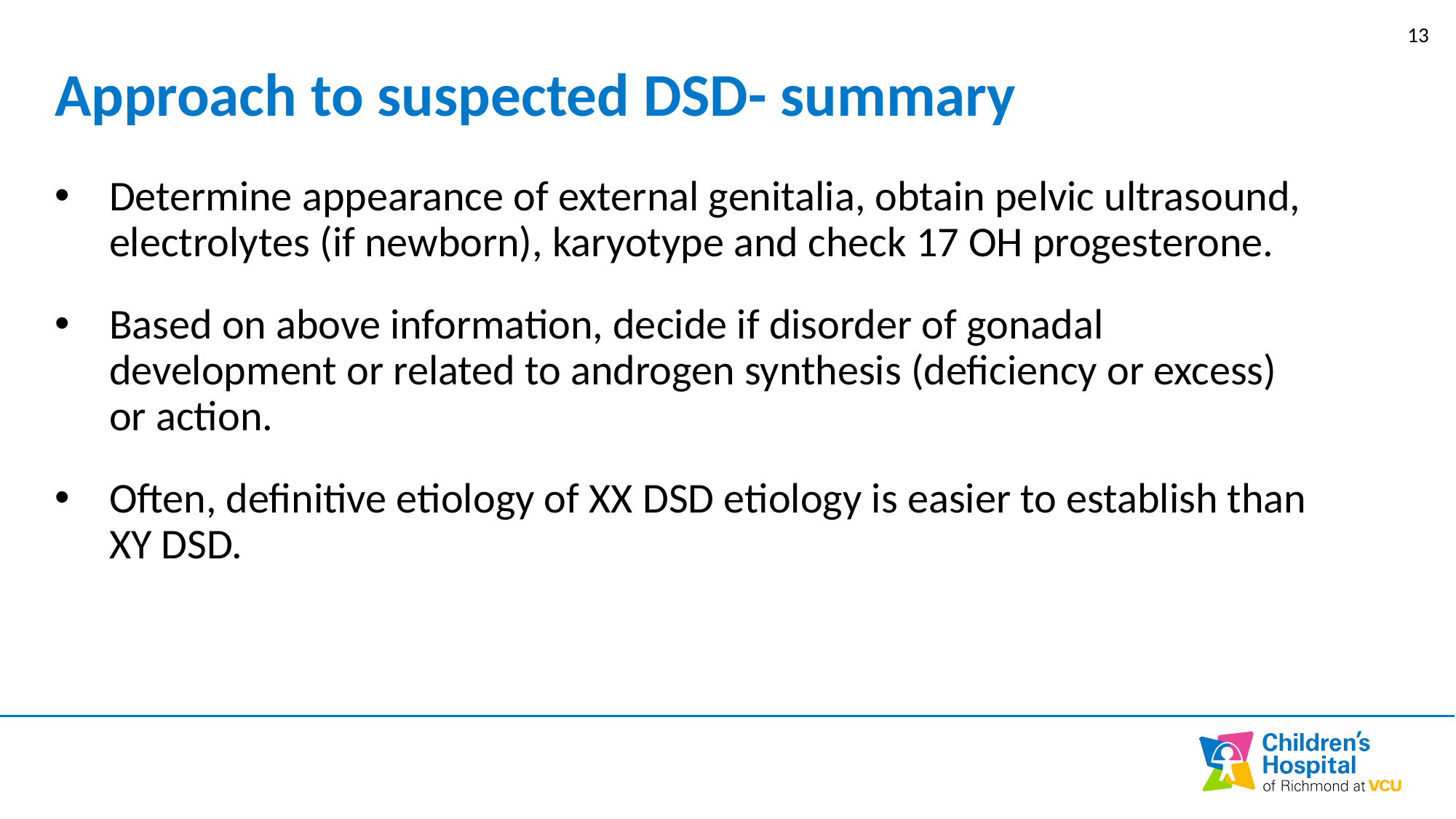

13
# Approach to suspected DSD- summary
Determine appearance of external genitalia, obtain pelvic ultrasound, electrolytes (if newborn), karyotype and check 17 OH progesterone.
Based on above information, decide if disorder of gonadal development or related to androgen synthesis (deficiency or excess) or action.
Often, definitive etiology of XX DSD etiology is easier to establish than XY DSD.

## Slide 14
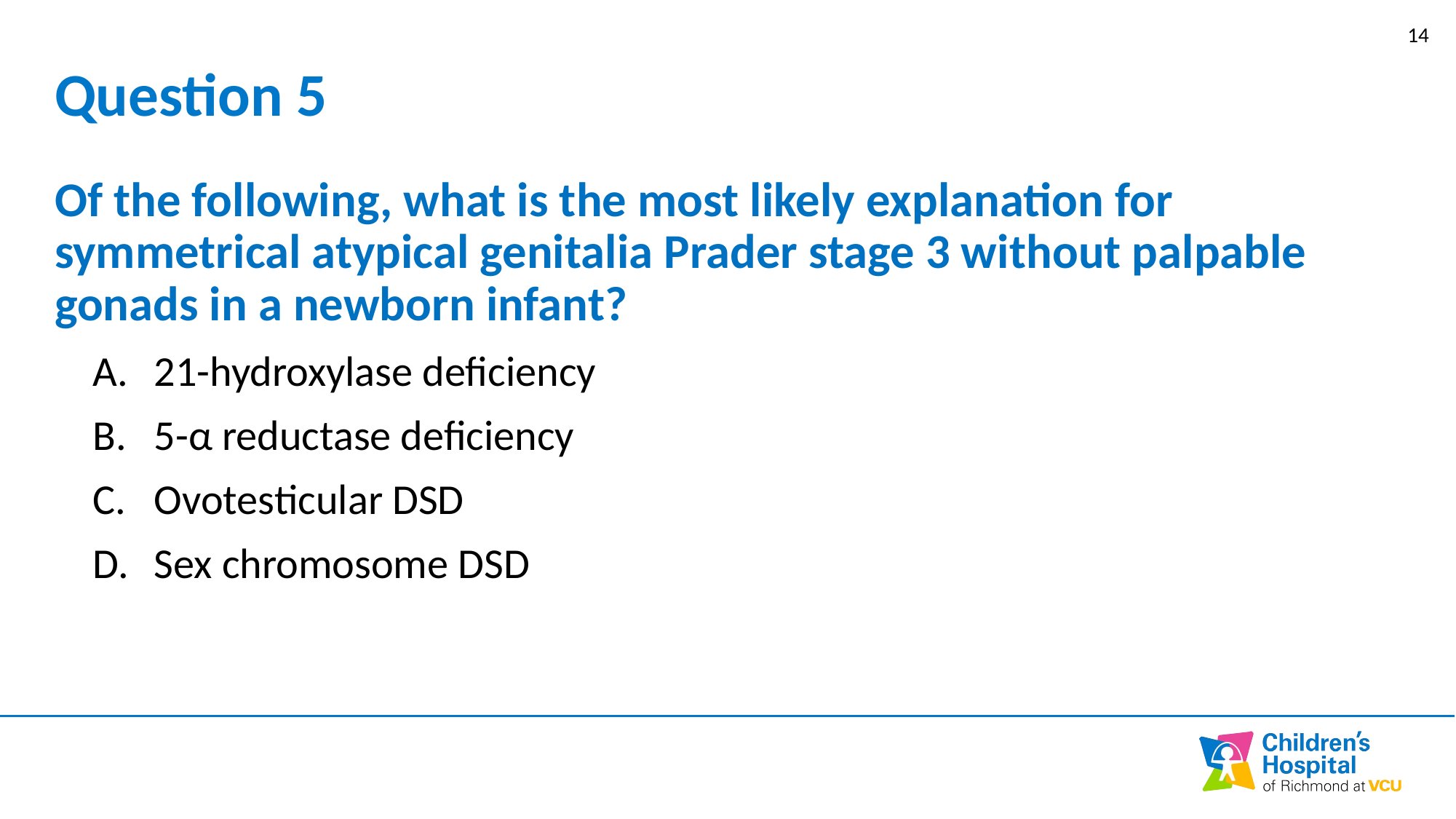

14
# Question 5
Of the following, what is the most likely explanation for symmetrical atypical genitalia Prader stage 3 without palpable gonads in a newborn infant?
21-hydroxylase deficiency
5-α reductase deficiency
Ovotesticular DSD
Sex chromosome DSD

## Slide 15
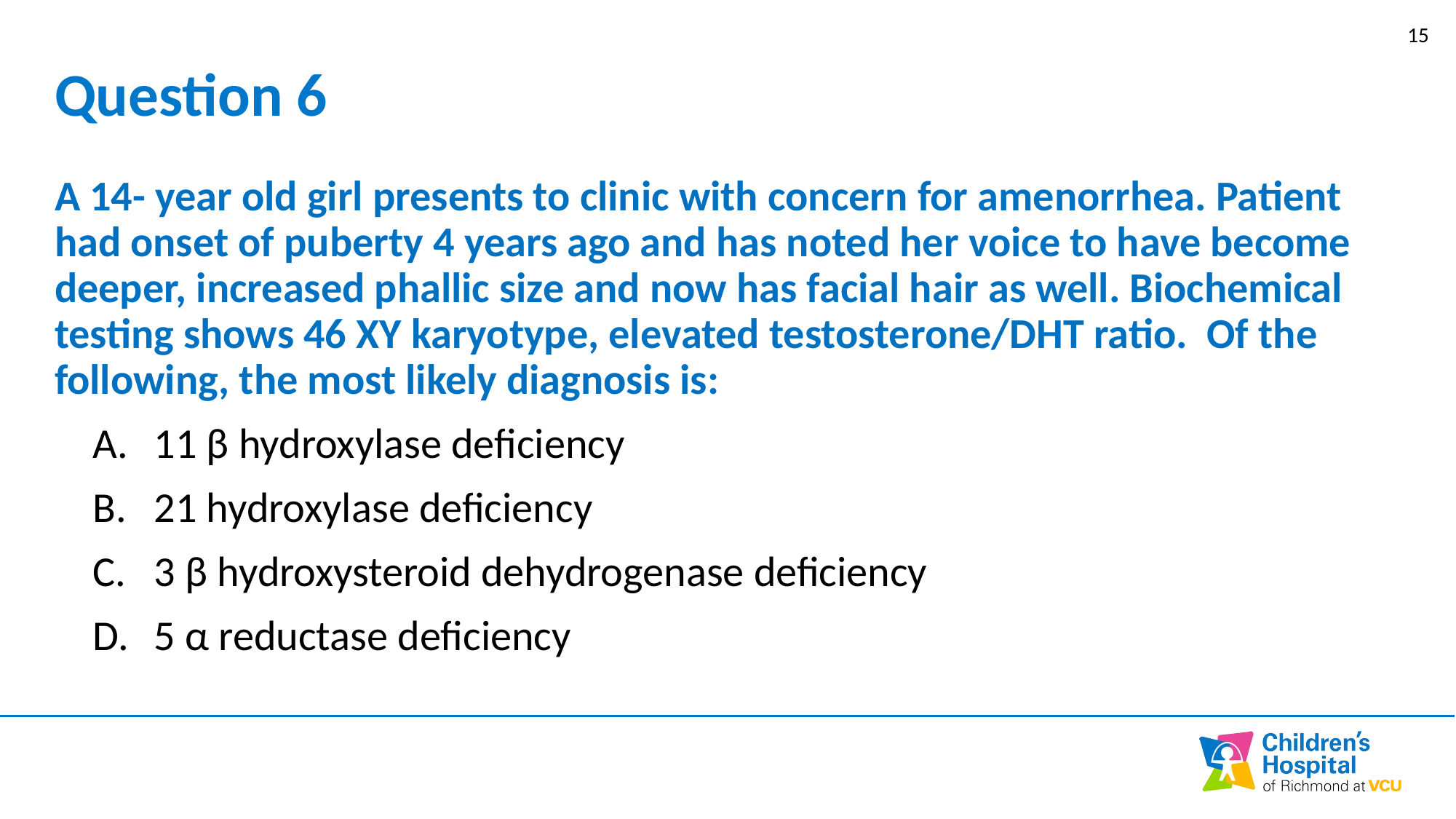

15
# Question 6
A 14- year old girl presents to clinic with concern for amenorrhea. Patient had onset of puberty 4 years ago and has noted her voice to have become deeper, increased phallic size and now has facial hair as well. Biochemical testing shows 46 XY karyotype, elevated testosterone/DHT ratio. Of the following, the most likely diagnosis is:
11 β hydroxylase deficiency
21 hydroxylase deficiency
3 β hydroxysteroid dehydrogenase deficiency
5 α reductase deficiency

## Slide 16
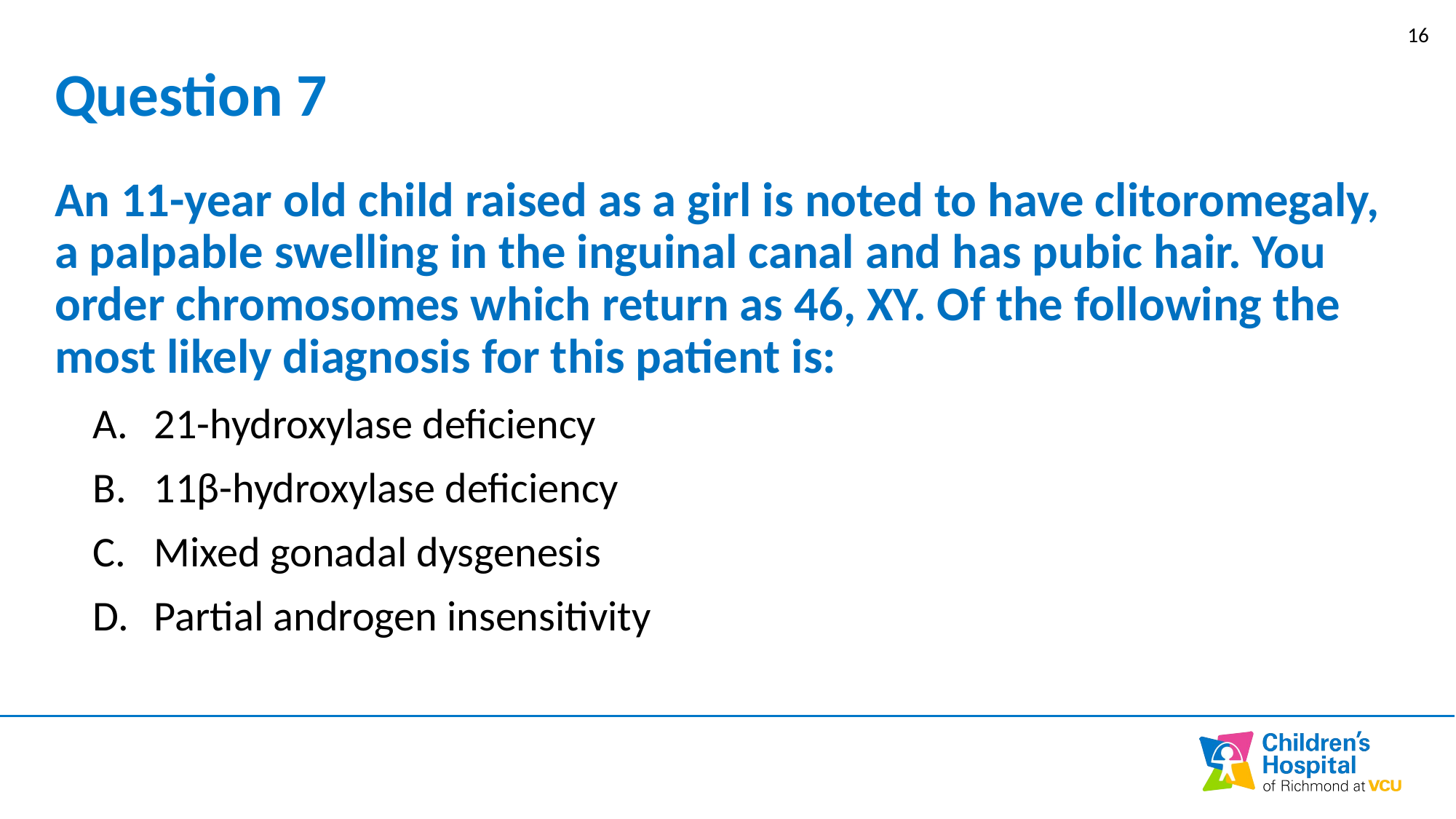

16
# Question 7
An 11-year old child raised as a girl is noted to have clitoromegaly, a palpable swelling in the inguinal canal and has pubic hair. You order chromosomes which return as 46, XY. Of the following the most likely diagnosis for this patient is:
21-hydroxylase deficiency
11β-hydroxylase deficiency
Mixed gonadal dysgenesis
Partial androgen insensitivity

## Slide 17
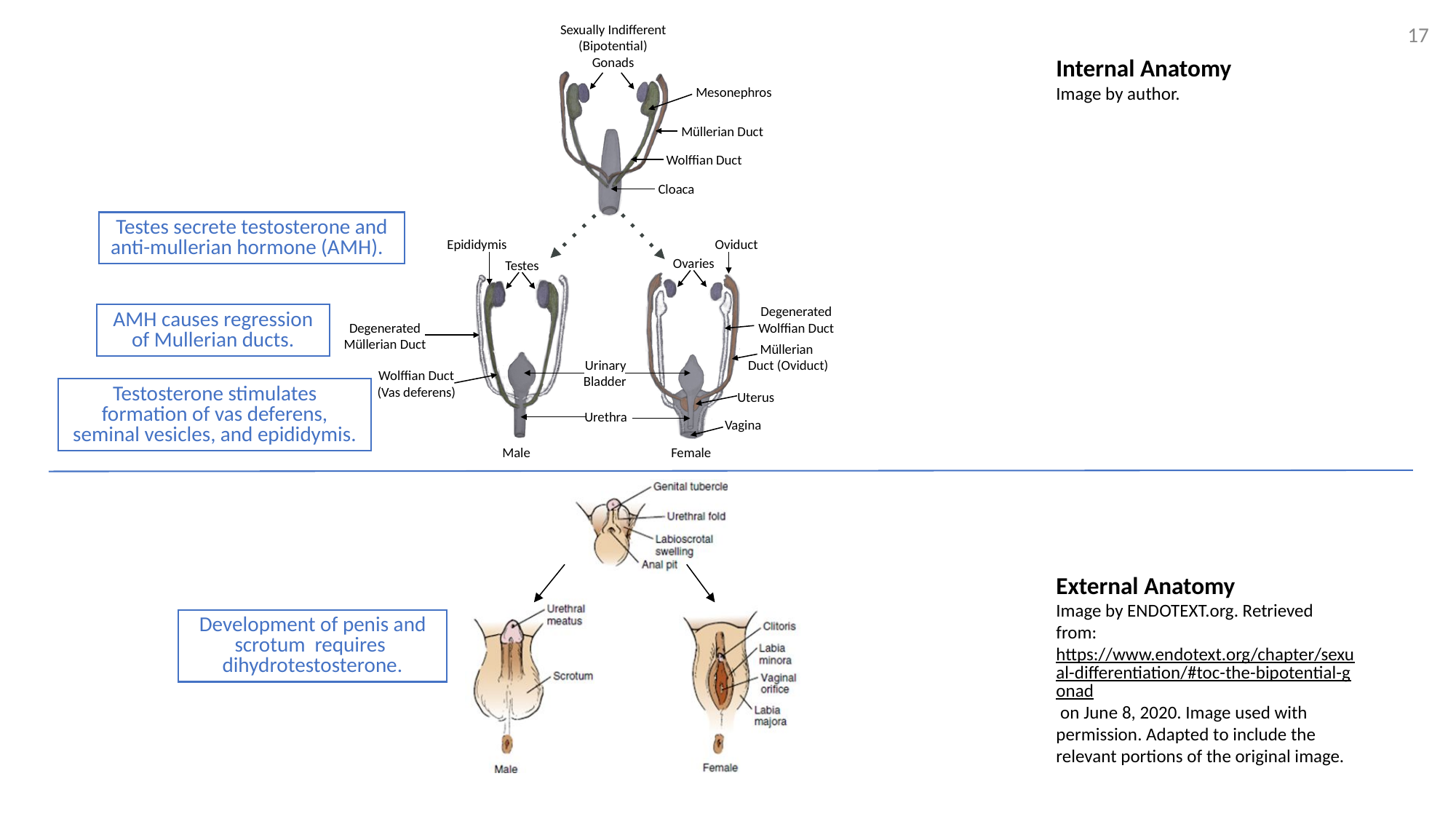

17
Sexually Indifferent
(Bipotential)
Gonads
Mesonephros
Müllerian Duct
Wolffian Duct
Cloaca
Epididymis
Oviduct
Ovaries
Testes
Degenerated Wolffian Duct
Degenerated Müllerian Duct
Müllerian
Duct (Oviduct)
Urinary Bladder
Wolffian Duct (Vas deferens)
Uterus
Urethra
Vagina
Male
Female
Internal Anatomy
Image by author.
Testes secrete testosterone and anti-mullerian hormone (AMH).
AMH causes regression of Mullerian ducts.
Testosterone stimulates formation of vas deferens, seminal vesicles, and epididymis.
External Anatomy
Image by ENDOTEXT.org. Retrieved from: https://www.endotext.org/chapter/sexual-differentiation/#toc-the-bipotential-gonad on June 8, 2020. Image used with permission. Adapted to include the relevant portions of the original image.
Development of penis and scrotum requires
dihydrotestosterone.

## Slide 18
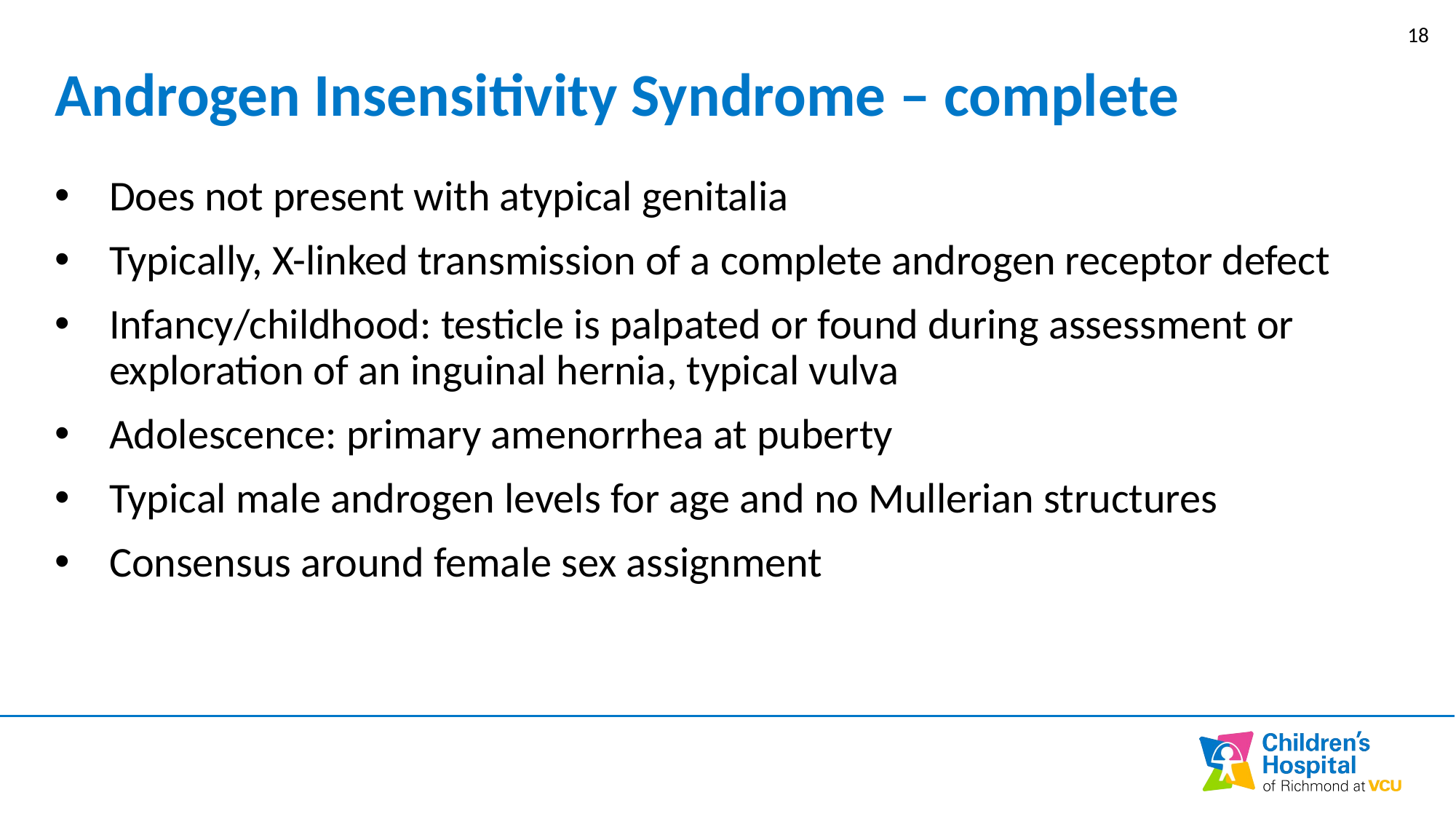

18
# Androgen Insensitivity Syndrome ‒ complete
Does not present with atypical genitalia
Typically, X-linked transmission of a complete androgen receptor defect
Infancy/childhood: testicle is palpated or found during assessment or exploration of an inguinal hernia, typical vulva
Adolescence: primary amenorrhea at puberty
Typical male androgen levels for age and no Mullerian structures
Consensus around female sex assignment

## Slide 19
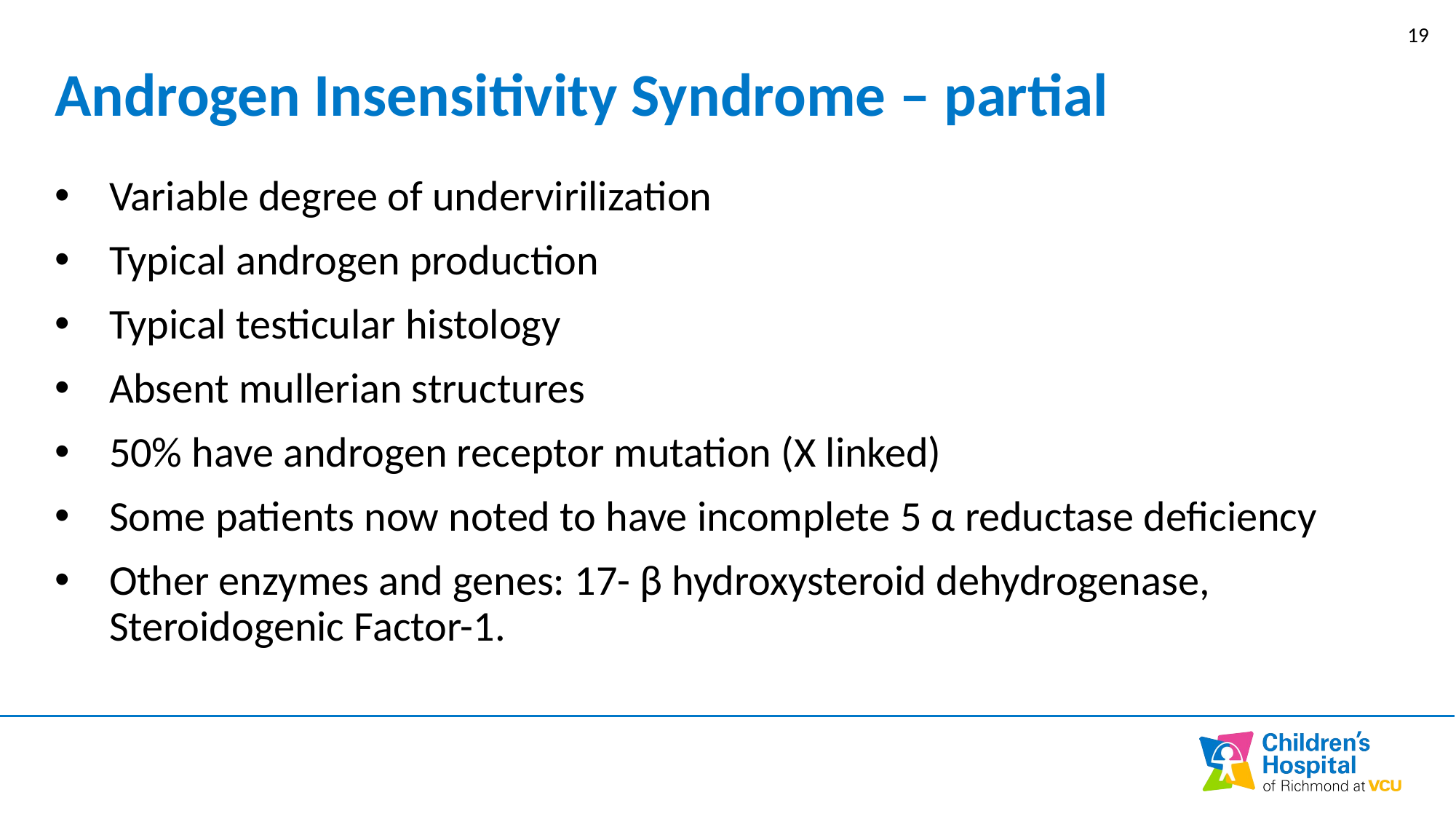

19
# Androgen Insensitivity Syndrome ‒ partial
Variable degree of undervirilization
Typical androgen production
Typical testicular histology
Absent mullerian structures
50% have androgen receptor mutation (X linked)
Some patients now noted to have incomplete 5 α reductase deficiency
Other enzymes and genes: 17- β hydroxysteroid dehydrogenase, Steroidogenic Factor-1.

## Slide 20
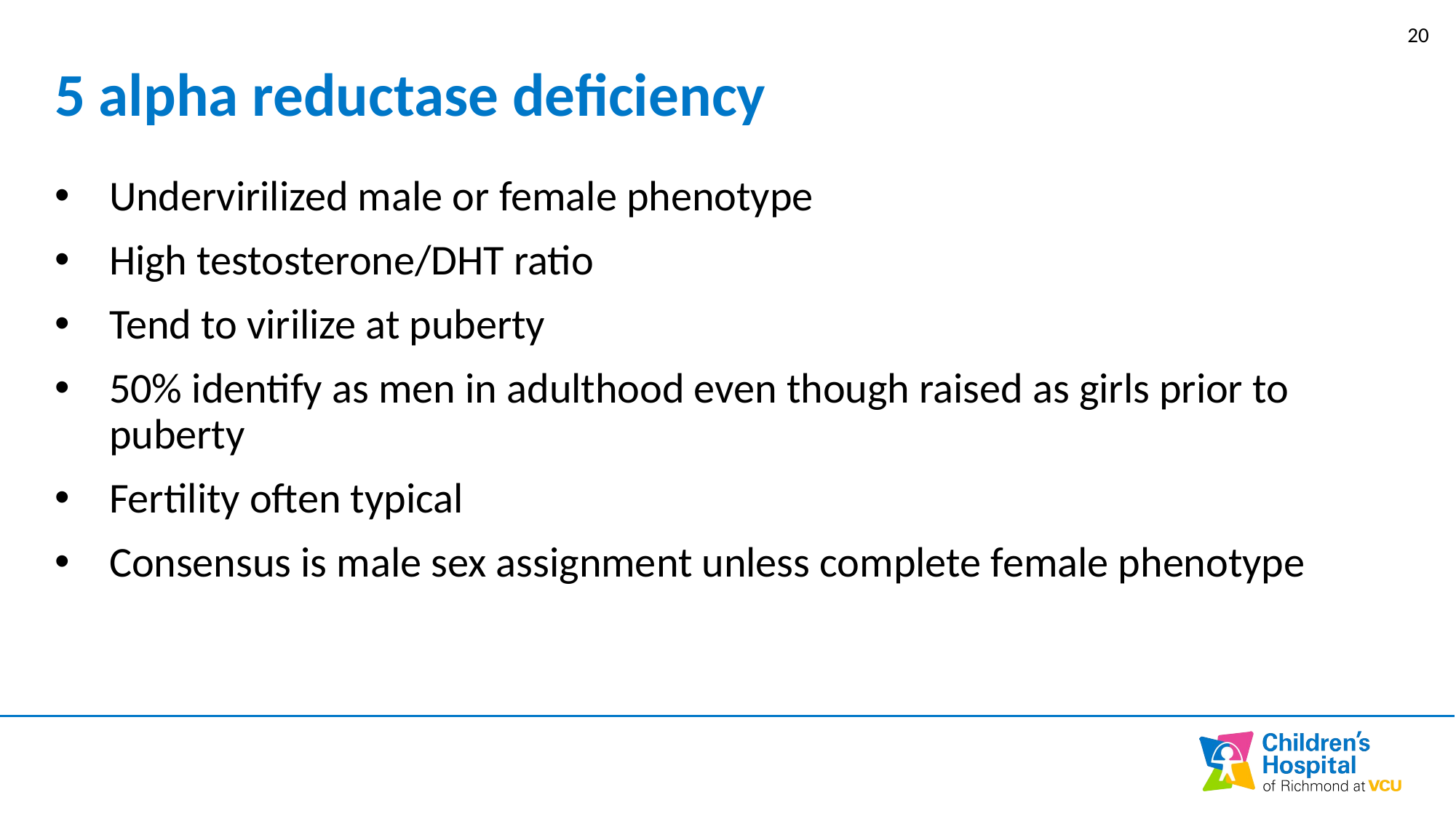

20
# 5 alpha reductase deficiency
Undervirilized male or female phenotype
High testosterone/DHT ratio
Tend to virilize at puberty
50% identify as men in adulthood even though raised as girls prior to puberty
Fertility often typical
Consensus is male sex assignment unless complete female phenotype

## Slide 21
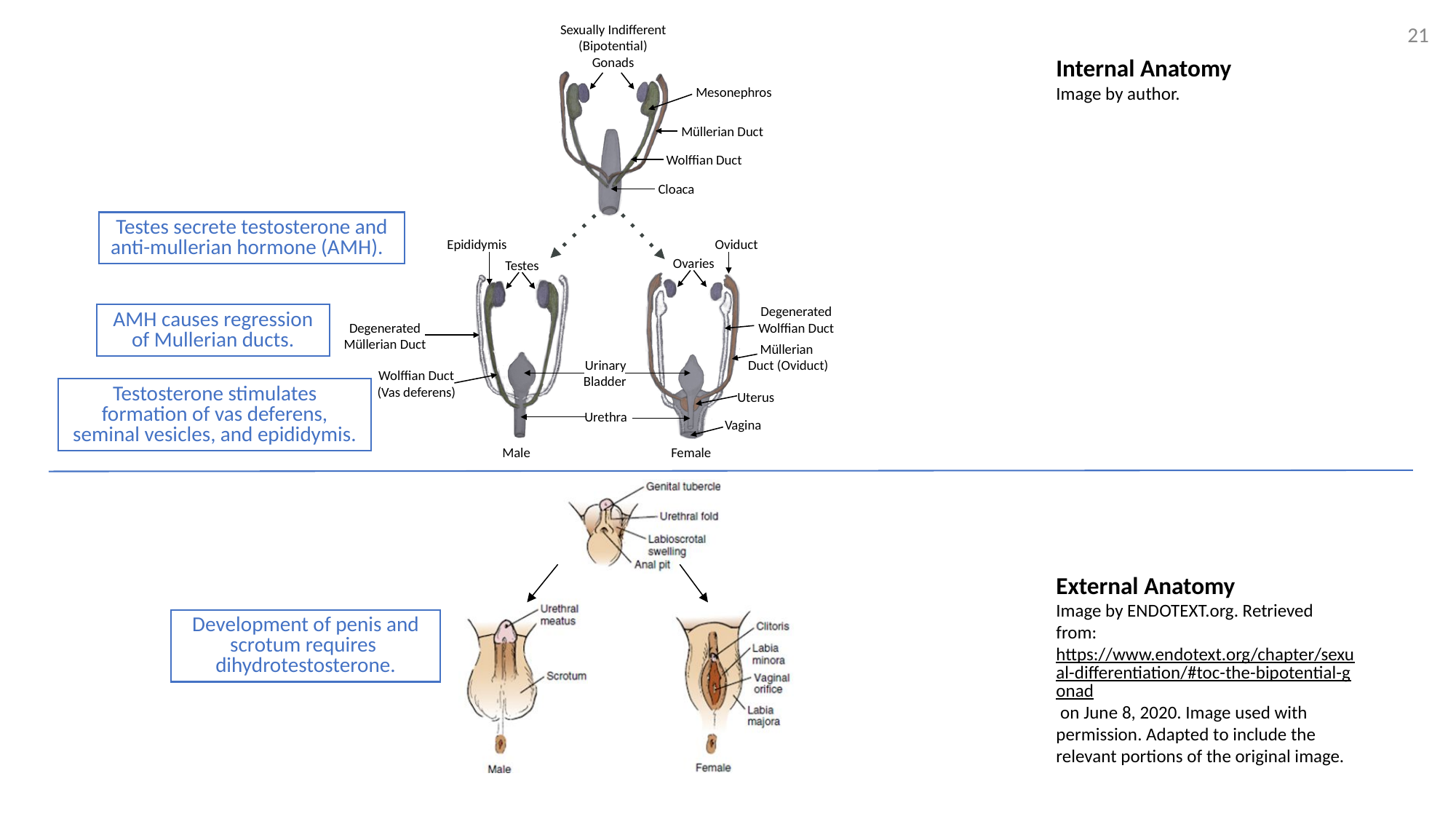

21
Sexually Indifferent
(Bipotential)
Gonads
Mesonephros
Müllerian Duct
Wolffian Duct
Cloaca
Epididymis
Oviduct
Ovaries
Testes
Degenerated Wolffian Duct
Degenerated Müllerian Duct
Müllerian
Duct (Oviduct)
Urinary Bladder
Wolffian Duct (Vas deferens)
Uterus
Urethra
Vagina
Male
Female
Internal Anatomy
Image by author.
Testes secrete testosterone and anti-mullerian hormone (AMH).
AMH causes regression of Mullerian ducts.
Testosterone stimulates formation of vas deferens, seminal vesicles, and epididymis.
External Anatomy
Image by ENDOTEXT.org. Retrieved from: https://www.endotext.org/chapter/sexual-differentiation/#toc-the-bipotential-gonad on June 8, 2020. Image used with permission. Adapted to include the relevant portions of the original image.
Development of penis and scrotum requires
dihydrotestosterone.

## Slide 22
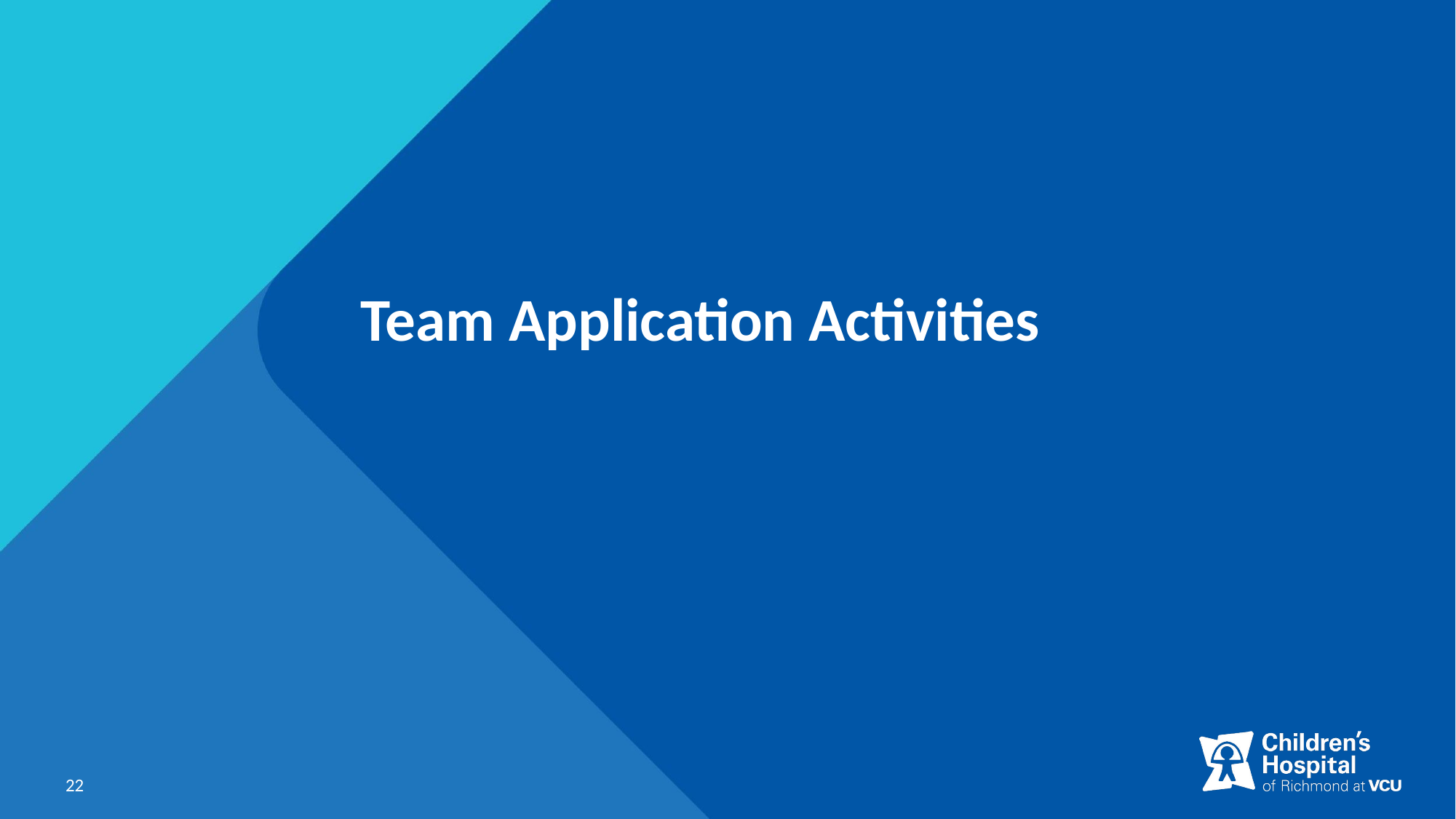

# Team Application Activities
22

## Slide 23
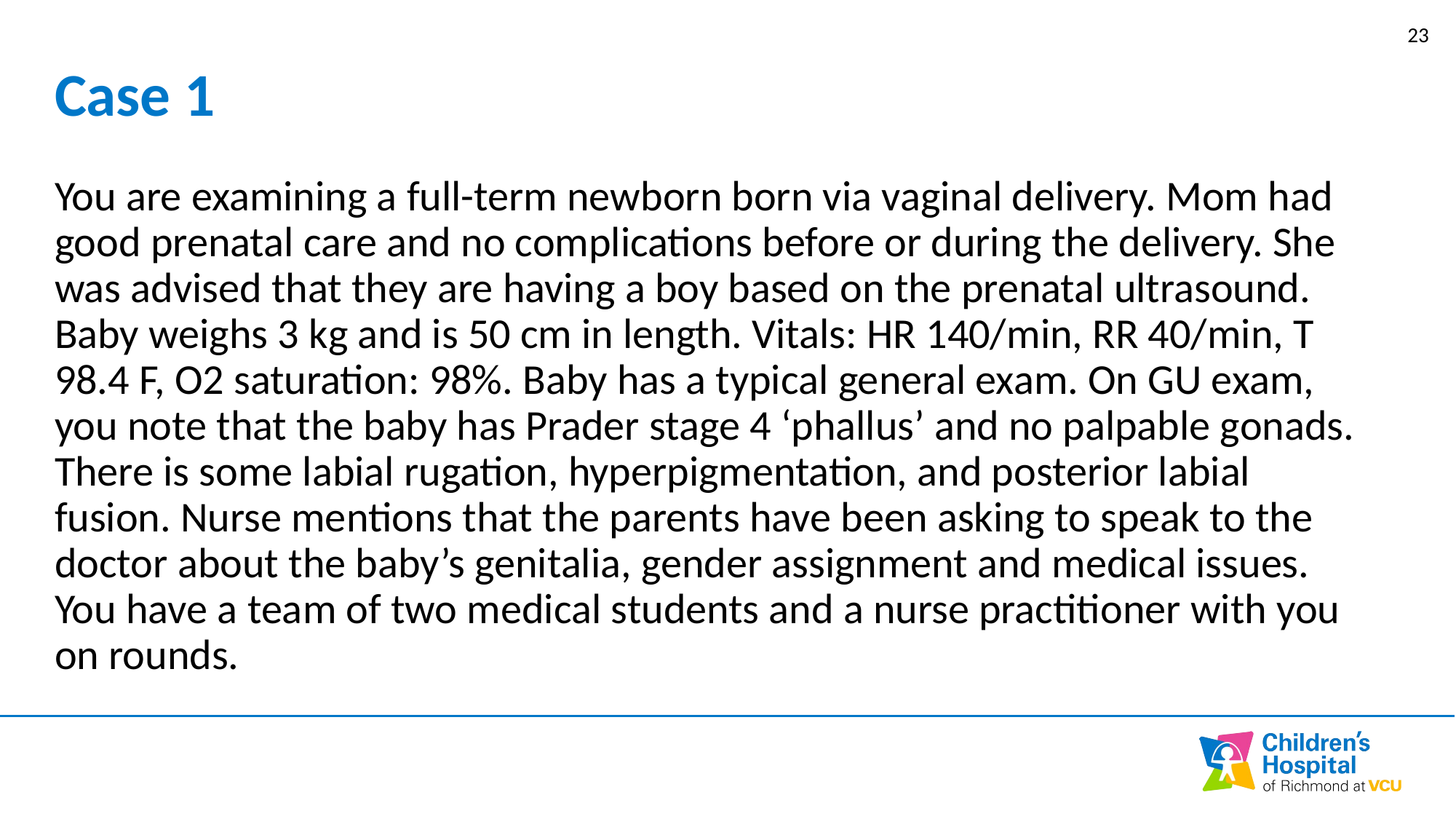

23
# Case 1
You are examining a full-term newborn born via vaginal delivery. Mom had good prenatal care and no complications before or during the delivery. She was advised that they are having a boy based on the prenatal ultrasound. Baby weighs 3 kg and is 50 cm in length. Vitals: HR 140/min, RR 40/min, T 98.4 F, O2 saturation: 98%. Baby has a typical general exam. On GU exam, you note that the baby has Prader stage 4 ‘phallus’ and no palpable gonads. There is some labial rugation, hyperpigmentation, and posterior labial fusion. Nurse mentions that the parents have been asking to speak to the doctor about the baby’s genitalia, gender assignment and medical issues. You have a team of two medical students and a nurse practitioner with you on rounds.

## Slide 24
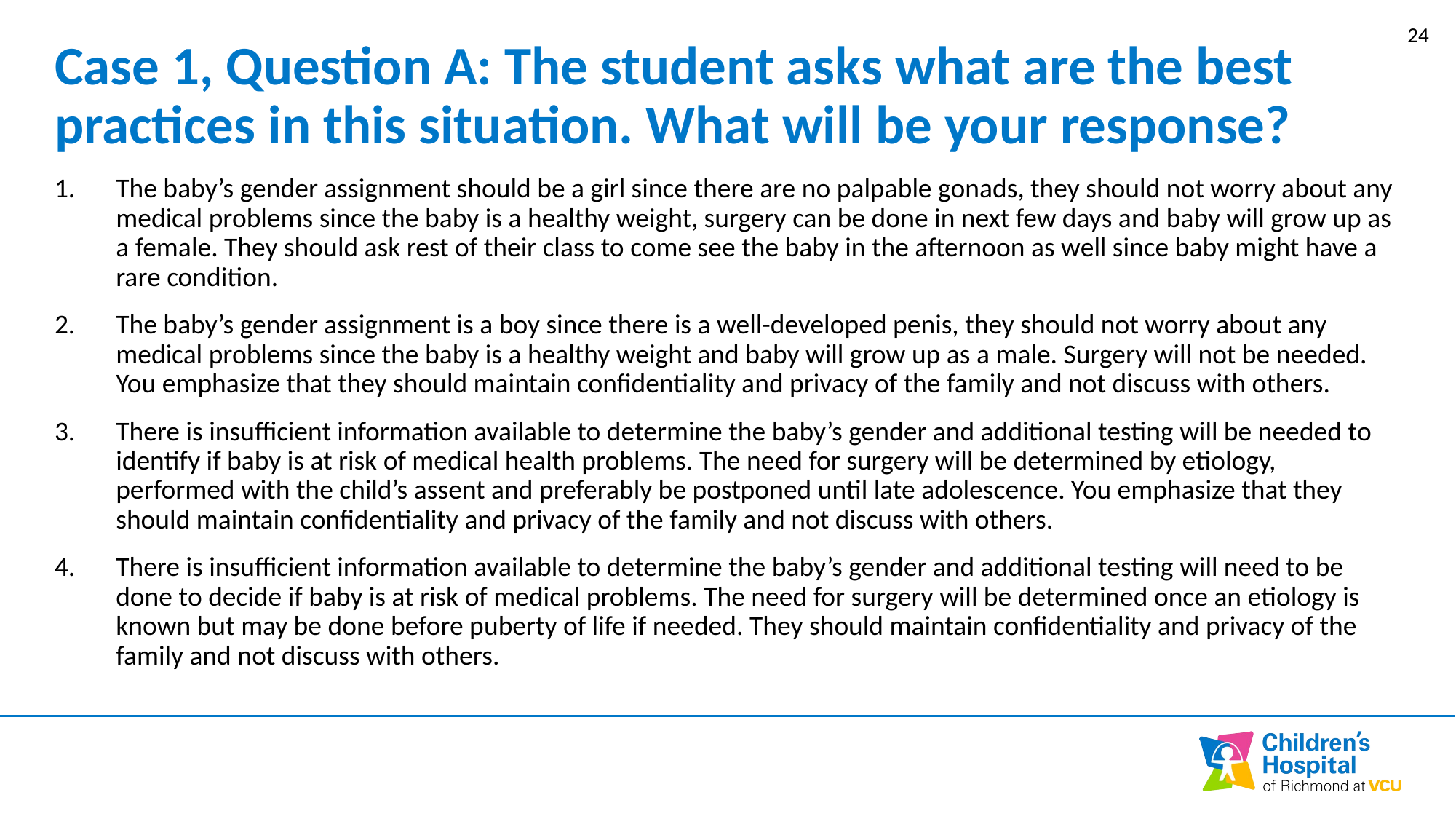

24
# Case 1, Question A: The student asks what are the best practices in this situation. What will be your response?
The baby’s gender assignment should be a girl since there are no palpable gonads, they should not worry about any medical problems since the baby is a healthy weight, surgery can be done in next few days and baby will grow up as a female. They should ask rest of their class to come see the baby in the afternoon as well since baby might have a rare condition.
The baby’s gender assignment is a boy since there is a well-developed penis, they should not worry about any medical problems since the baby is a healthy weight and baby will grow up as a male. Surgery will not be needed. You emphasize that they should maintain confidentiality and privacy of the family and not discuss with others.
There is insufficient information available to determine the baby’s gender and additional testing will be needed to identify if baby is at risk of medical health problems. The need for surgery will be determined by etiology, performed with the child’s assent and preferably be postponed until late adolescence. You emphasize that they should maintain confidentiality and privacy of the family and not discuss with others.
There is insufficient information available to determine the baby’s gender and additional testing will need to be done to decide if baby is at risk of medical problems. The need for surgery will be determined once an etiology is known but may be done before puberty of life if needed. They should maintain confidentiality and privacy of the family and not discuss with others.

## Slide 25
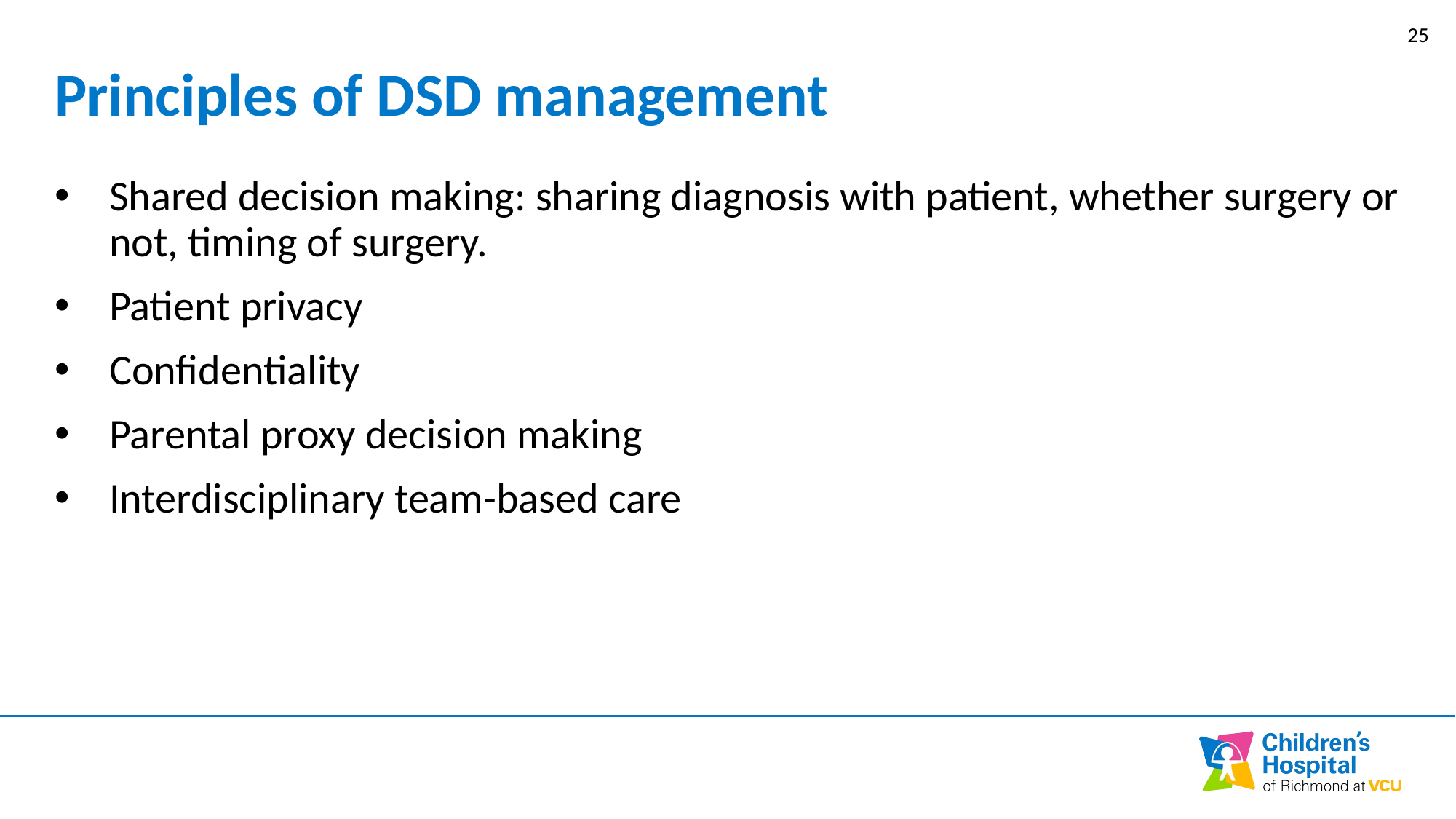

25
# Principles of DSD management
Shared decision making: sharing diagnosis with patient, whether surgery or not, timing of surgery.
Patient privacy
Confidentiality
Parental proxy decision making
Interdisciplinary team-based care

## Slide 26
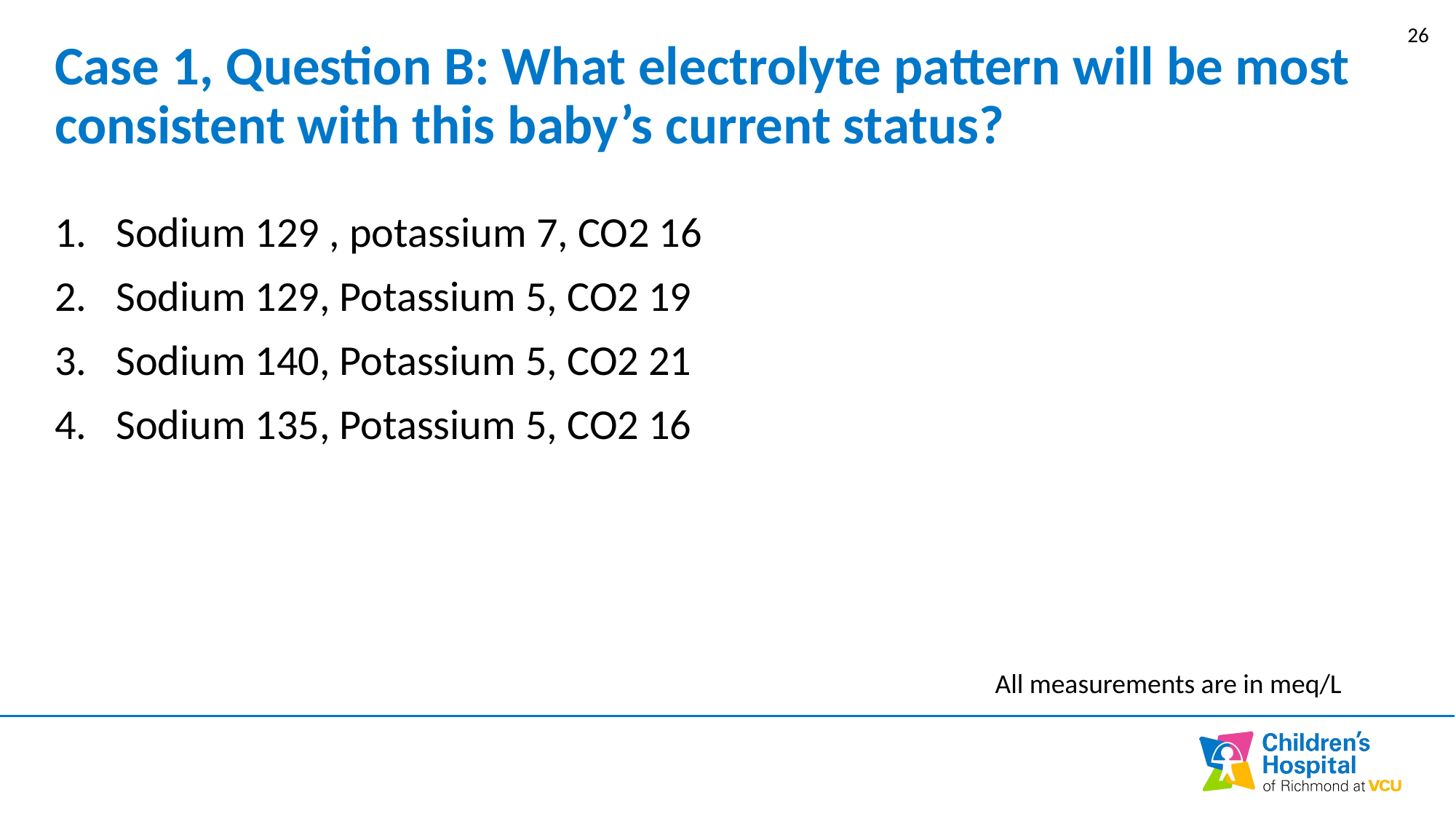

26
# Case 1, Question B: What electrolyte pattern will be most consistent with this baby’s current status?
Sodium 129 , potassium 7, CO2 16
Sodium 129, Potassium 5, CO2 19
Sodium 140, Potassium 5, CO2 21
Sodium 135, Potassium 5, CO2 16
All measurements are in meq/L

## Slide 27
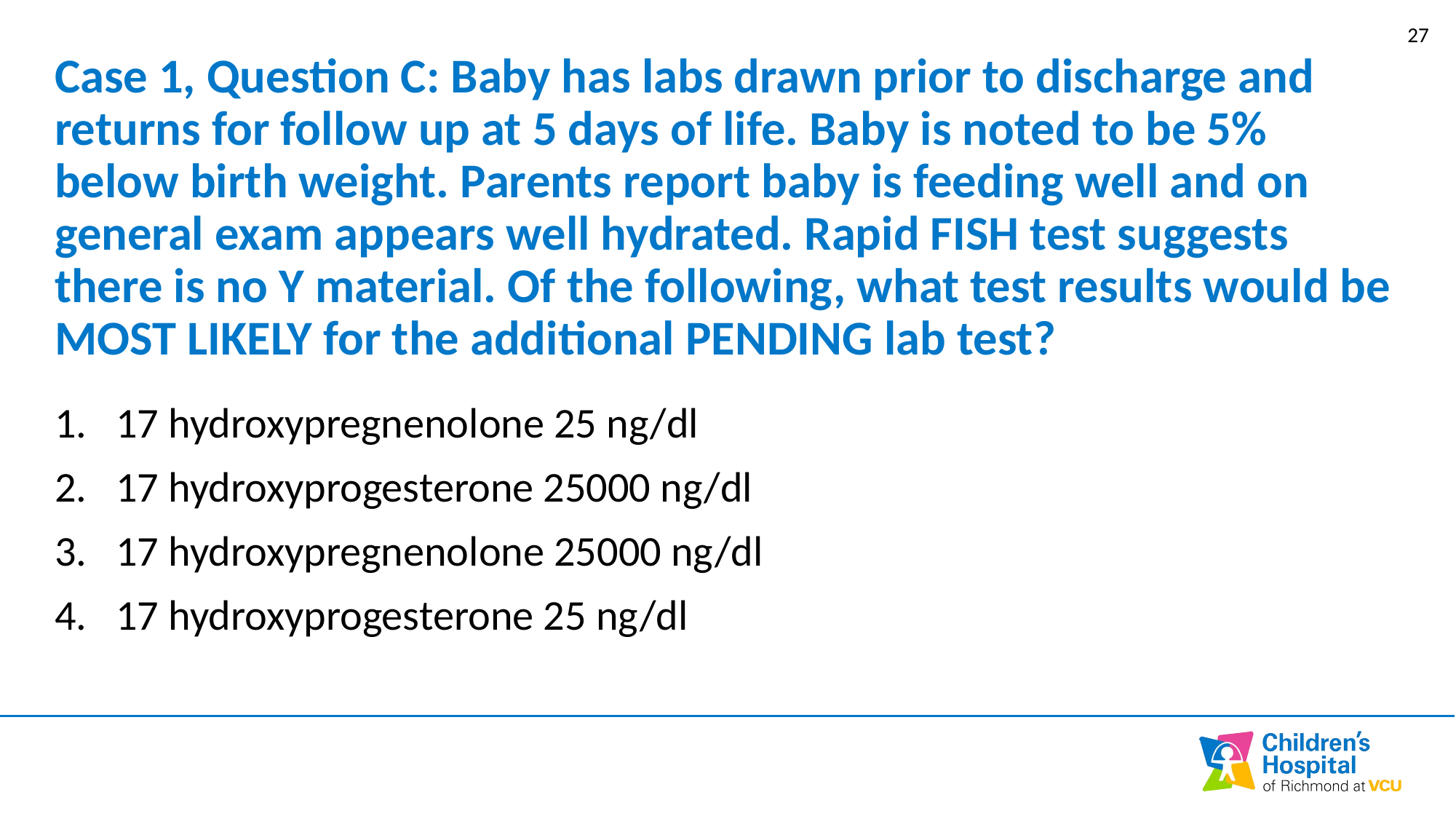

27
# Case 1, Question C: Baby has labs drawn prior to discharge and returns for follow up at 5 days of life. Baby is noted to be 5% below birth weight. Parents report baby is feeding well and on general exam appears well hydrated. Rapid FISH test suggests there is no Y material. Of the following, what test results would be MOST LIKELY for the additional PENDING lab test?
17 hydroxypregnenolone 25 ng/dl
17 hydroxyprogesterone 25000 ng/dl
17 hydroxypregnenolone 25000 ng/dl
17 hydroxyprogesterone 25 ng/dl

## Slide 28
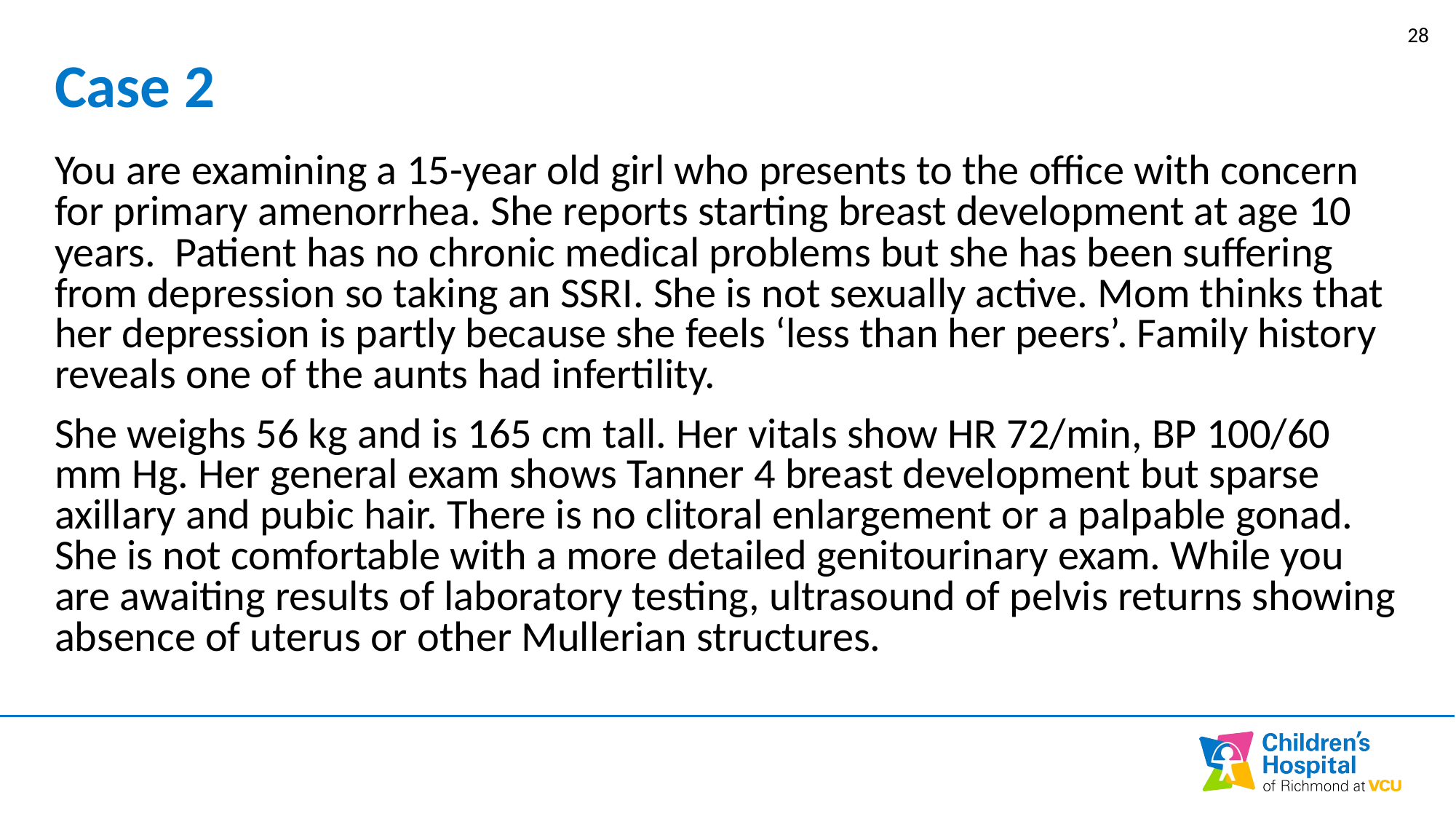

28
# Case 2
You are examining a 15-year old girl who presents to the office with concern for primary amenorrhea. She reports starting breast development at age 10 years. Patient has no chronic medical problems but she has been suffering from depression so taking an SSRI. She is not sexually active. Mom thinks that her depression is partly because she feels ‘less than her peers’. Family history reveals one of the aunts had infertility.
She weighs 56 kg and is 165 cm tall. Her vitals show HR 72/min, BP 100/60 mm Hg. Her general exam shows Tanner 4 breast development but sparse axillary and pubic hair. There is no clitoral enlargement or a palpable gonad. She is not comfortable with a more detailed genitourinary exam. While you are awaiting results of laboratory testing, ultrasound of pelvis returns showing absence of uterus or other Mullerian structures.

## Slide 29
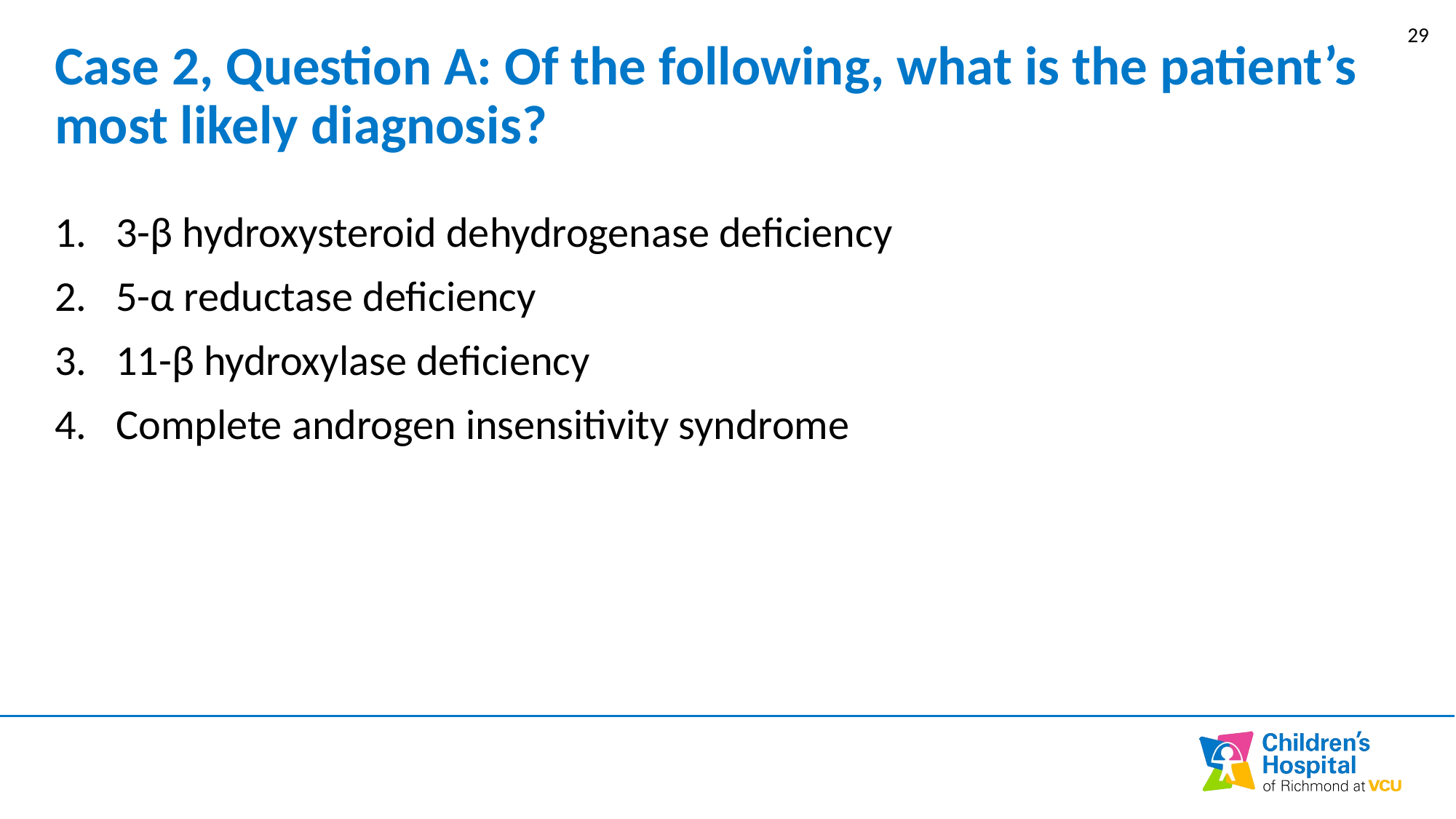

29
# Case 2, Question A: Of the following, what is the patient’s most likely diagnosis?
3-β hydroxysteroid dehydrogenase deficiency
5-α reductase deficiency
11-β hydroxylase deficiency
Complete androgen insensitivity syndrome

## Slide 30
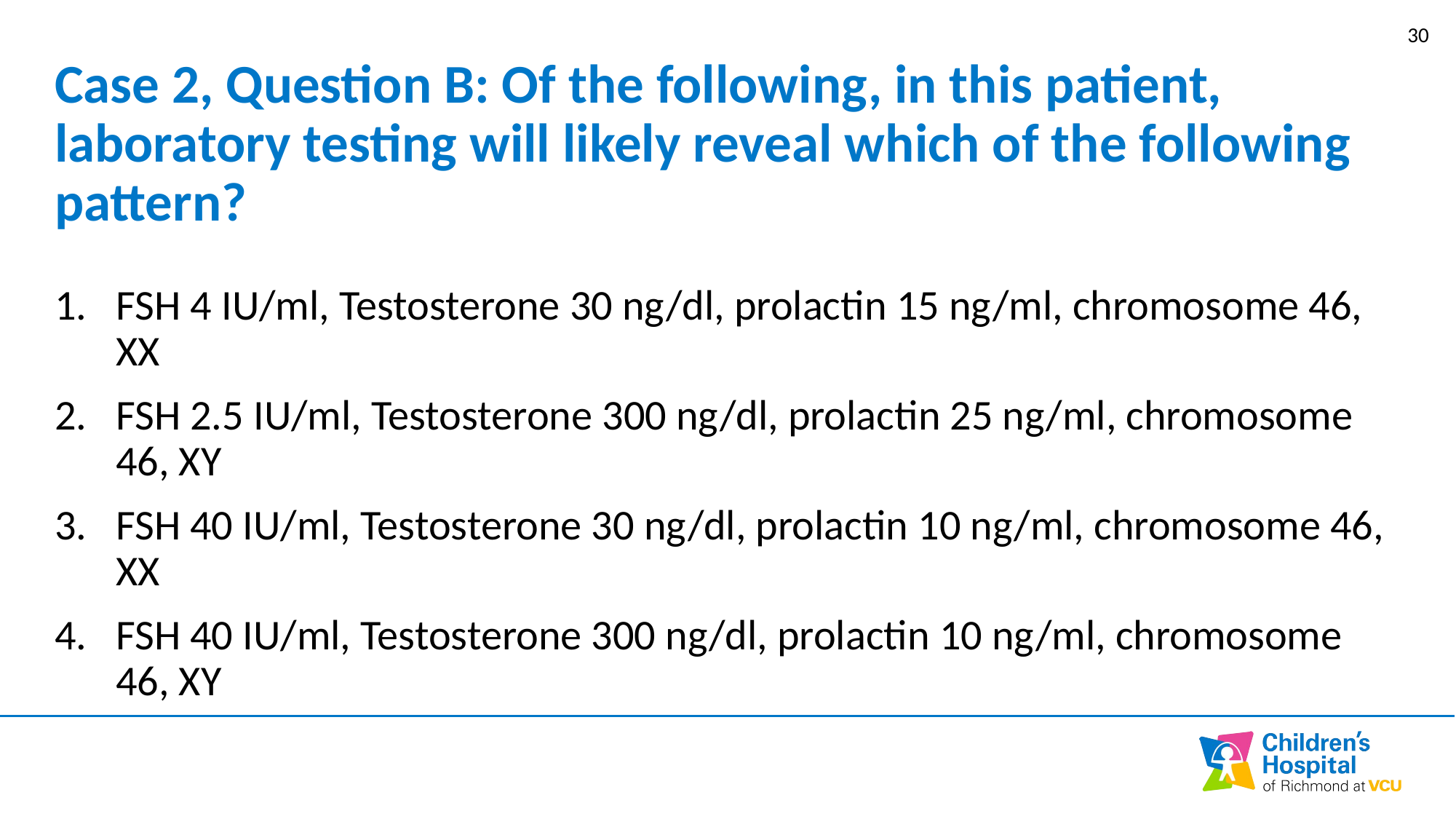

30
# Case 2, Question B: Of the following, in this patient, laboratory testing will likely reveal which of the following pattern?
FSH 4 IU/ml, Testosterone 30 ng/dl, prolactin 15 ng/ml, chromosome 46, XX
FSH 2.5 IU/ml, Testosterone 300 ng/dl, prolactin 25 ng/ml, chromosome 46, XY
FSH 40 IU/ml, Testosterone 30 ng/dl, prolactin 10 ng/ml, chromosome 46, XX
FSH 40 IU/ml, Testosterone 300 ng/dl, prolactin 10 ng/ml, chromosome 46, XY

## Slide 31
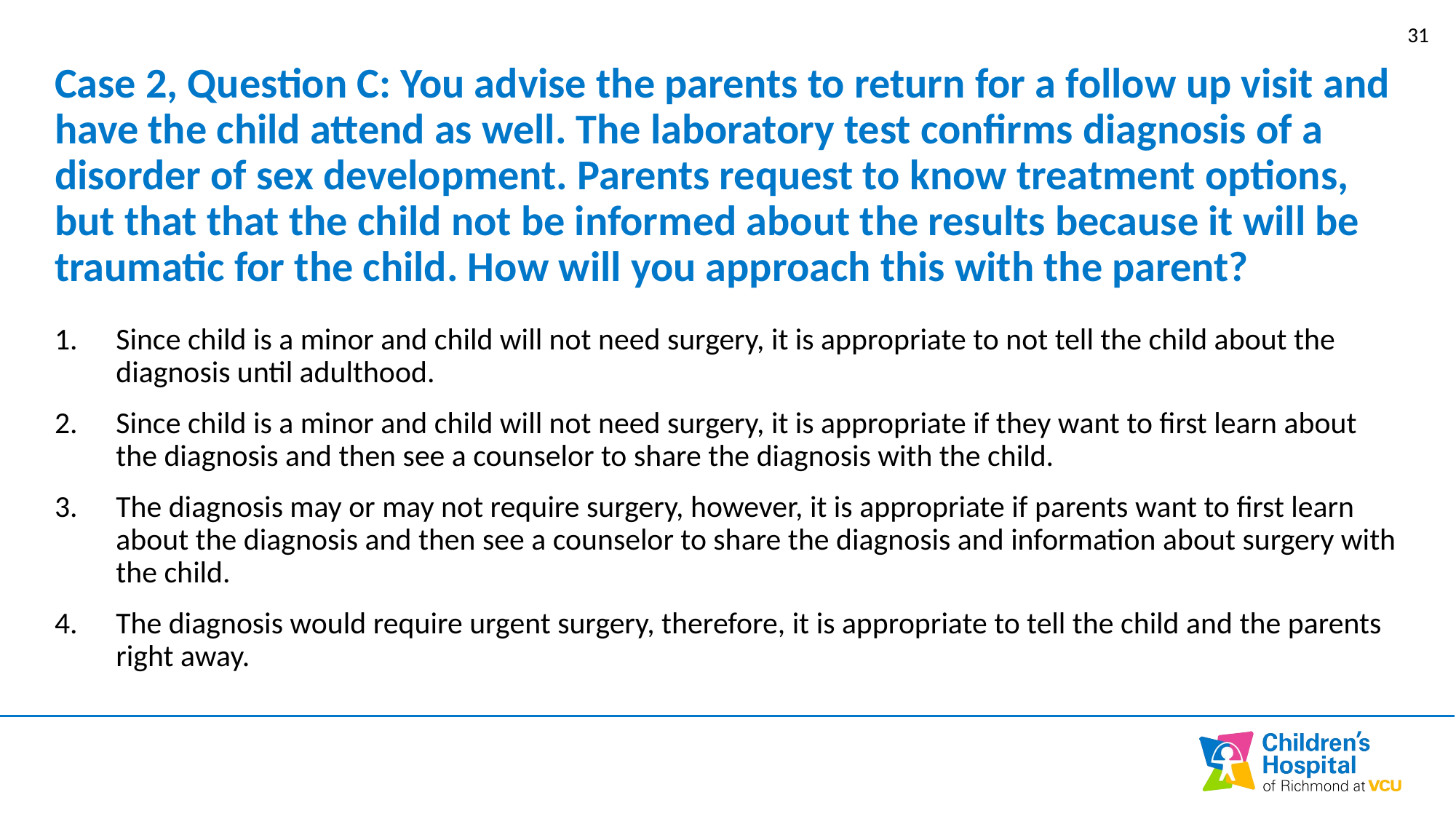

31
# Case 2, Question C: You advise the parents to return for a follow up visit and have the child attend as well. The laboratory test confirms diagnosis of a disorder of sex development. Parents request to know treatment options, but that that the child not be informed about the results because it will be traumatic for the child. How will you approach this with the parent?
Since child is a minor and child will not need surgery, it is appropriate to not tell the child about the diagnosis until adulthood.
Since child is a minor and child will not need surgery, it is appropriate if they want to first learn about the diagnosis and then see a counselor to share the diagnosis with the child.
The diagnosis may or may not require surgery, however, it is appropriate if parents want to first learn about the diagnosis and then see a counselor to share the diagnosis and information about surgery with the child.
The diagnosis would require urgent surgery, therefore, it is appropriate to tell the child and the parents right away.

## Slide 32
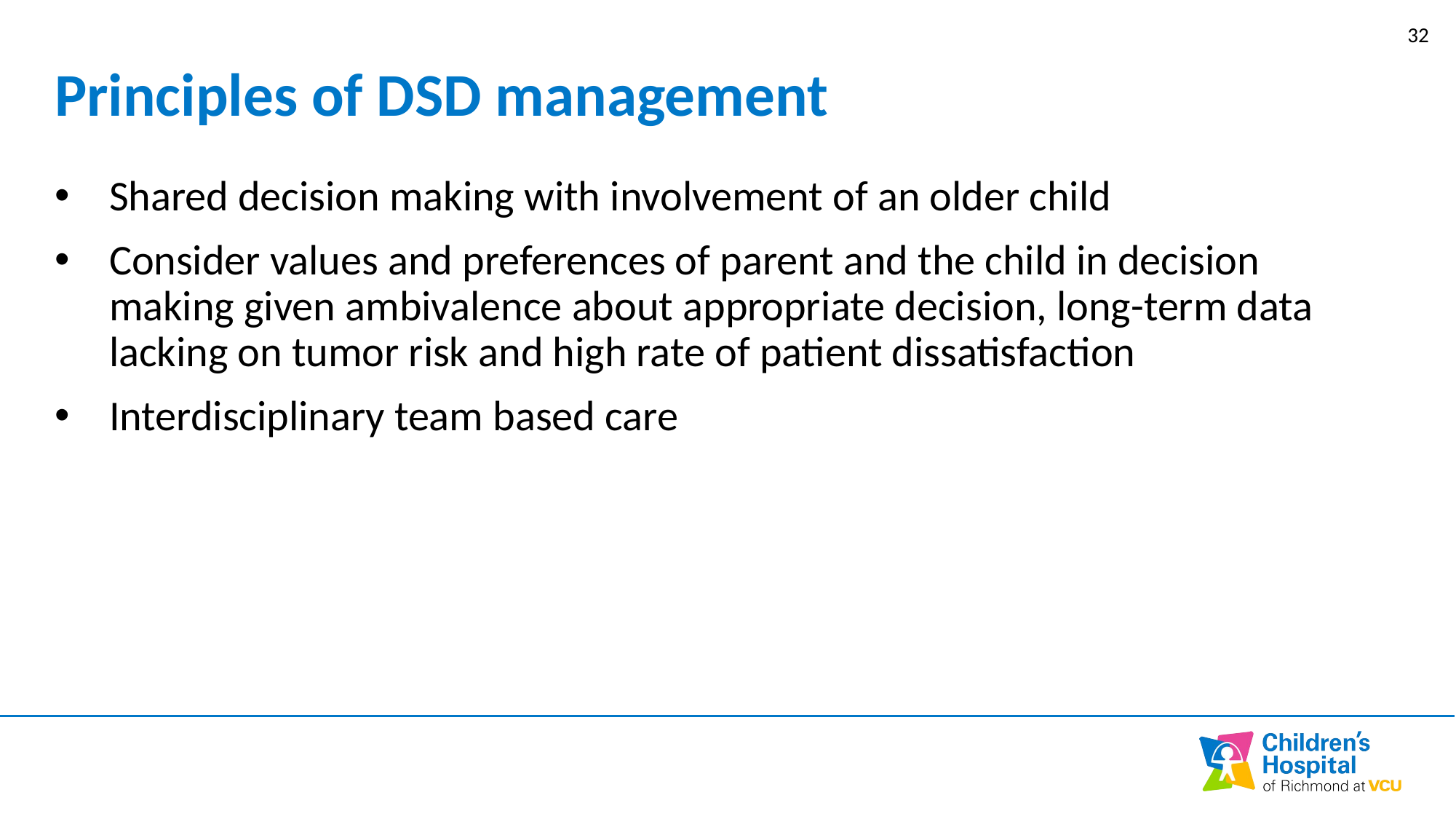

32
# Principles of DSD management
Shared decision making with involvement of an older child
Consider values and preferences of parent and the child in decision making given ambivalence about appropriate decision, long-term data lacking on tumor risk and high rate of patient dissatisfaction
Interdisciplinary team based care

## Slide 33
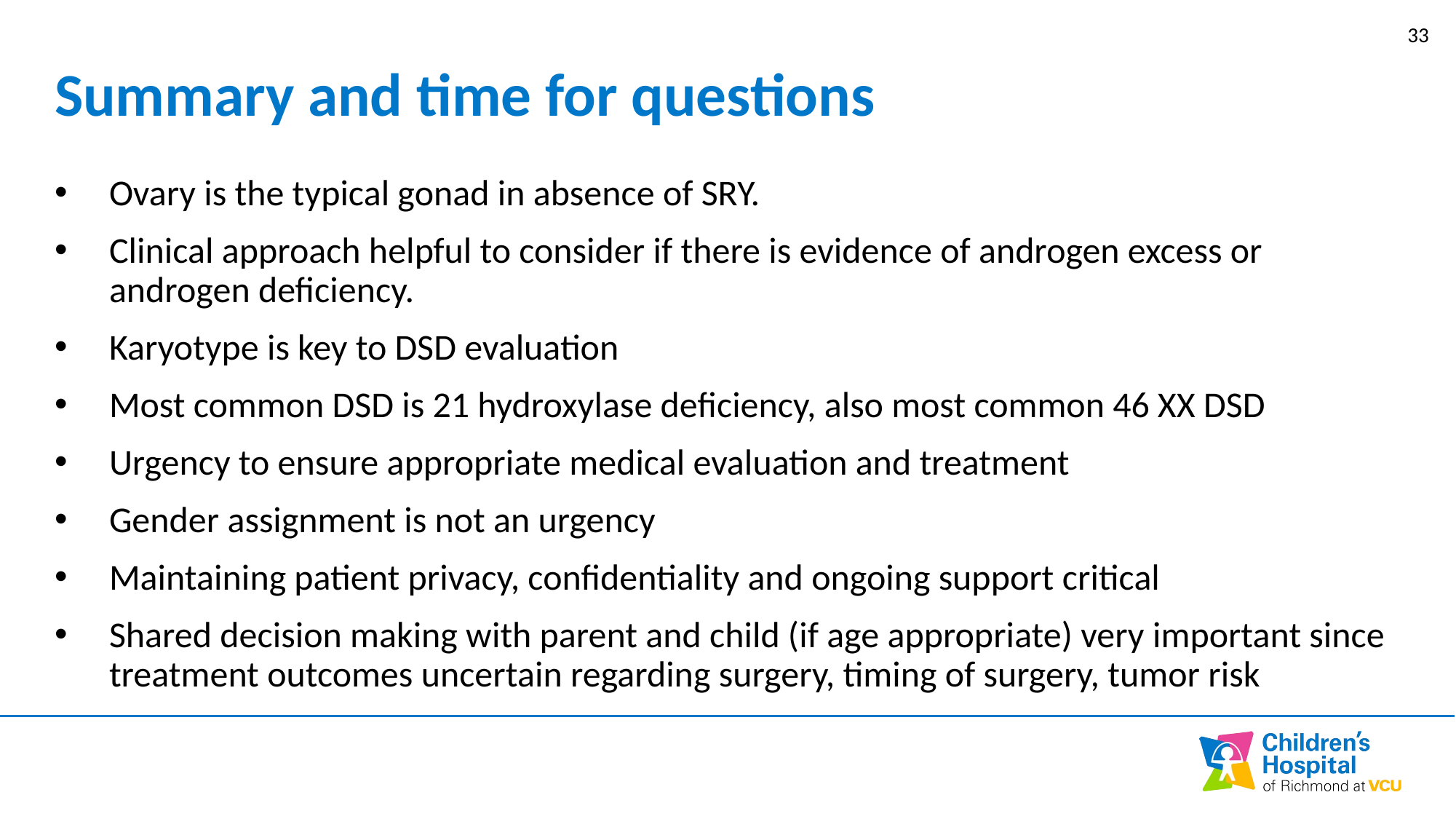

33
# Summary and time for questions
Ovary is the typical gonad in absence of SRY.
Clinical approach helpful to consider if there is evidence of androgen excess or androgen deficiency.
Karyotype is key to DSD evaluation
Most common DSD is 21 hydroxylase deficiency, also most common 46 XX DSD
Urgency to ensure appropriate medical evaluation and treatment
Gender assignment is not an urgency
Maintaining patient privacy, confidentiality and ongoing support critical
Shared decision making with parent and child (if age appropriate) very important since treatment outcomes uncertain regarding surgery, timing of surgery, tumor risk

## Slide 34
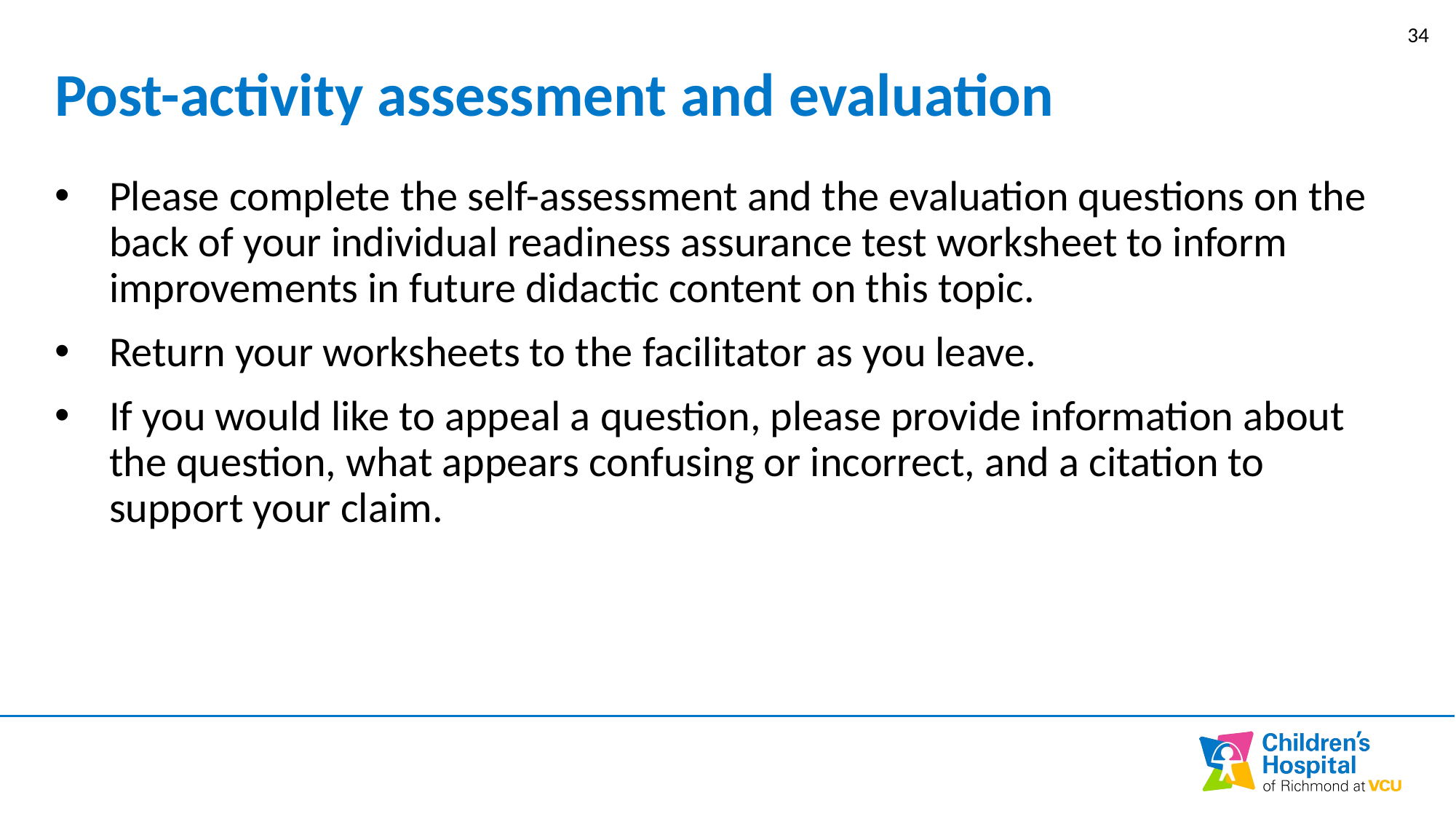

34
# Post-activity assessment and evaluation
Please complete the self-assessment and the evaluation questions on the back of your individual readiness assurance test worksheet to inform improvements in future didactic content on this topic.
Return your worksheets to the facilitator as you leave.
If you would like to appeal a question, please provide information about the question, what appears confusing or incorrect, and a citation to support your claim.

## Slide 35
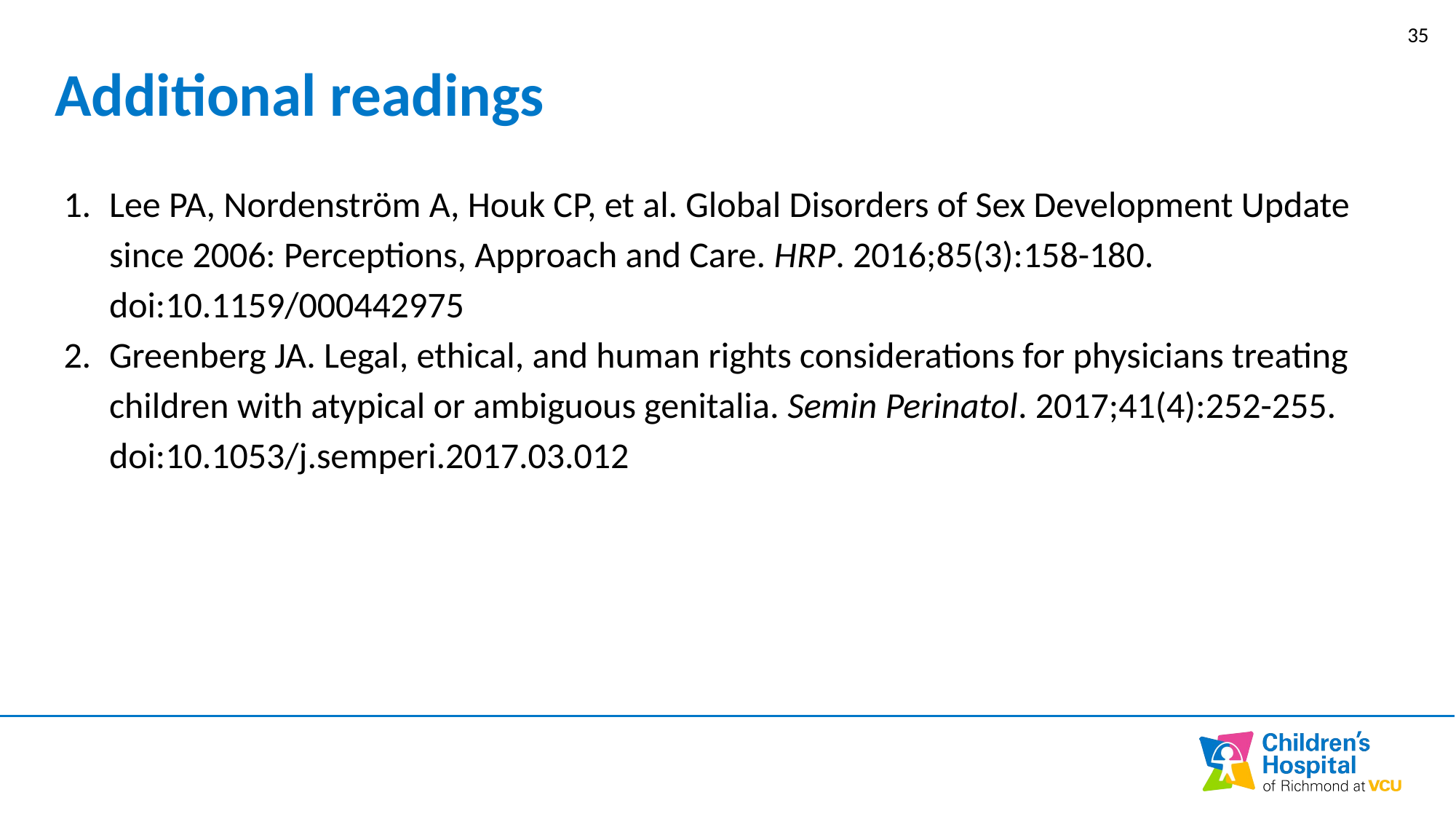

35
# Additional readings
Lee PA, Nordenström A, Houk CP, et al. Global Disorders of Sex Development Update since 2006: Perceptions, Approach and Care. HRP. 2016;85(3):158-180. doi:10.1159/000442975
Greenberg JA. Legal, ethical, and human rights considerations for physicians treating children with atypical or ambiguous genitalia. Semin Perinatol. 2017;41(4):252-255. doi:10.1053/j.semperi.2017.03.012
