# Supplementary material for: Increasing Knowledge and Self-Efficacy on Differences in Sex Development (DSD): A Team-Based Learning Activity for Pediatric Residents
Source: MedEdPORTAL. 2021 Feb 23;17:11105. doi: 10.15766/mep_2374-8265.11105 (PMC7901252; doi:10.15766/mep_2374-8265.11105)
Supplement: Supplementary file 1 — Team Materials List.docxPre-Post Assessment iRAT Response Form.docxTBL Activity Slides.pptxStudent RAT.docxFacilitator RAT.docxFacilitator Team Application Activity.docxStudent Team Application Activity.docxAdrenal Enzyme Pathway Diagram.docxPrader Scale Handout.docx [file mep_2374-8265.11105-s001.zip › D. Student RAT.docx]

ATTENTION, STUDENTS: If you are accessing this material BEFORE it is used in your course, please do NOT read this document prior to the class session. This activity is designed to lead you through a learning experience that reinforces your knowledge of the content. Early review or dissemination of this material to others will diminish the learning opportunity and be considered academic misconduct.

Differences in Sex Development (DSD):
Team-Based Learning Module for Pediatric Residents

**Readiness Assurance Test**

1. What is/are the main driving factor/s for development of bipotential gonad into a testicle?
   1. Pituitary gland hormones
   2. Sex determining region in the Y chromosome
   3. Sex determining region in the X chromosome
   4. Sex steroid hormones
2. What is a clinical scenario that raises clinical suspicion for DSD?
   1. Enlarged clitoral hood without posterior labial fusion
   2. Enlarged clitoris with posterior labial fusion
   3. First degree hypospadias with unilateral undescended testes
   4. Second degree hypospadias with descended testes
3. What aspects of maternal or family medical history should be actively inquired into when evaluating a child with atypical genitalia?
   1. Diabetes mellitus in father
   2. Hyperthyroidism in mother
   3. Irregular menses in a relative
   4. Premature birth in sister
4. Of the following, what is the most IMPORTANT test in evaluation of a six-month old with one palpable gonad and perineo-scrotal hypospadias?
   1. Androstenedione
   2. Karyotype
   3. Progesterone
   4. Urethrogram
5. Of the following, what is the most likely explanation for symmetrical atypical genitalia with Prader stage 3 without palpable gonads in a newborn infant?
   1. 21 hydroxylase deficiency
   2. 5 alpha reductase deficiency
   3. Ovotesticular DSD
   4. Sex chromosome DSD
6. A 14-year old girl presents to clinic with concern for amenorrhea. Patient had onset of puberty 4 years ago and has noted her voice to have become deeper, increased phallic size and now has facial hair as well. Biochemical testing shows 46 XY karyotype, elevated testosterone/DHT ratio. Of the following, the most likely diagnosis is:
   1. 11 beta hydroxylase deficiency
   2. 21 hydroxylase deficiency
   3. 3-beta hydroxysteroid dehydrogenase deficiency
   4. 5 alpha reductase deficiency
7. An 11-year old child raised as a girl is noted to have clitoromegaly (Prader 3), a palpable swelling in the inguinal canal and has pubic hair. Chromosomes show 46, XY pattern. Of the following the most likely diagnosis for this patient is:
   1. 21 hydroxylase deficiency
   2. 11-beta hydroxylase deficiency
   3. Mixed gonadal dysgenesis
   4. Partial androgen insensitivity
