# Supplementary material for: Increasing Knowledge and Self-Efficacy on Differences in Sex Development (DSD): A Team-Based Learning Activity for Pediatric Residents
Source: MedEdPORTAL. 2021 Feb 23;17:11105. doi: 10.15766/mep_2374-8265.11105 (PMC7901252; doi:10.15766/mep_2374-8265.11105)
Supplement: Supplementary file 1 — Team Materials List.docxPre-Post Assessment iRAT Response Form.docxTBL Activity Slides.pptxStudent RAT.docxFacilitator RAT.docxFacilitator Team Application Activity.docxStudent Team Application Activity.docxAdrenal Enzyme Pathway Diagram.docxPrader Scale Handout.docx [file mep_2374-8265.11105-s001.zip › E. Facilitator RAT.docx]

ATTENTION, STUDENTS: If you are accessing this material BEFORE it is used in your course, please do NOT read this document prior to the class session. An answer key is included in this module, which is designed to lead you through a learning experience that reinforces your knowledge of the content. Early review or dissemination of this material to others will diminish the learning opportunity and be considered academic misconduct.

**FACILITATOR VERSION**

Differences in Sex Development (DSD):
Team-Based Learning Module for Pediatric Residents

**Readiness Assurance Test**

1. What is/are the main driving factor/s for development of bipotential gonad into a testicle?
   1. Pituitary gland hormones
   2. Sex determining region in the Y chromosome
   3. Sex determining region in the X chromosome
   4. Sex steroid hormones

*Facilitators: What is the time period when gonads are undifferentiated? What is the default gonad? Which genes are critical for differentiation of bipotential gonad into testes? (SRY gene in the Y chromosome)*

1. What is a clinical scenario that raises clinical suspicion for DSD?
   1. Enlarged clitoral hood without posterior labial fusion
   2. Enlarged clitoris with posterior labial fusion
   3. First degree hypospadias with unilateral undescended testes
   4. Second degree hypospadias with descended testes

*Facilitators: The presence of features of androgen excess in an infant with appearance of female vulva (clitoral enlargement Prader 3 or more, posterior labial fusion, urogenital sinus ) or of androgen deficiency in an infant with male appearing genitals (bilateral undescended testes with perineo-scrotal hypospadias, bifid scrotum, micropenis) should raise suspicion for DSD and need for evaluation. Isolated clitoromegaly, first/second degree hypospadias are not typically associated with DSD.*

1. What aspects of maternal or family medical history should be actively inquired into when evaluating a child with atypical genitalia?
   1. Diabetes mellitus in father
   2. Hyperthyroidism in mother
   3. Irregular menses in a relative
   4. Premature birth in sister

*Facilitators: While it is important to establish maternal and family medical history, often families might not be aware of details of a family history of DSD, it is important to actively ask about history of hypospadias, amenorrhea and infertility in family members and it need not be present in first degree relatives. Prematurity, paternal diabetes or hyperthyroidism in a first degree relative have not been associated with risk of DSD.*

1. Of the following, what is the most IMPORTANT test in evaluation of a six-month old with one palpable gonad and perineo-scrotal hypospadias?
   1. Androstenedione
   2. Karyotype
   3. Progesterone
   4. Urethrogram

*Facilitators: A karyotype is a good starting point to differentiate between 46XX, 46XY or 45X/46XY DSD. While 17-hydroxyprogesterone is important to check in suspected DSD related to 21 hydroxylase deficiency, there is no role of progesterone. Since patient may not be in minipuberty, gonadotropins may not be very informative even if low. Androstenedione is a precursor to testosterone so may be helpful in subsequent evaluation but not as an initial step in above scenario.*

1. Of the following, what is the most likely explanation for symmetrical atypical genitalia with Prader stage 3 without palpable gonads in a newborn infant?
   1. 21 hydroxylase deficiency
   2. 5 alpha reductase deficiency
   3. Ovotesticular DSD
   4. Sex chromosome DSD

*Facilitators: The most common etiology of a newborn with DSD with symmetrical atypical genitalia without palpable gonads is an appearance of androgen excess, most common being 21-hydroxylase deficiency. XX gonadal dysgenesis can rarely present with atypical genitalia if Y material present but not common. Disorders due to XY DSD may either have gonadal dysgenesis or a disorder of androgen synthesis/action. Complete (dysgenesis or enzyme defect) will not present with atypical genitalia; these disorders may present later in life with primary amenorrhea. Partial dysgenesis or enzyme defects present with variable degree of androgen deficiency and can present as symmetrical atypical genitalia without palpable gonads at birth. Sex chromosome DSD usually present with no ambiguity (classic Turner or Klinefelter syndrome) or with asymmetrical atypical genitalia (45,X/46,XY). Ovotesticular DSD is uncommon and will typically present as asymmetrical atypical genitalia.*

1. A 14-year old girl presents to clinic with concern for amenorrhea. Patient had onset of puberty 4 years ago and has noted her voice to have become deeper, increased phallic size and now has facial hair as well. Biochemical testing shows 46 XY karyotype, elevated testosterone/DHT ratio. Of the following, the most likely diagnosis is:
   1. 11 beta hydroxylase deficiency
   2. 21 hydroxylase deficiency
   3. 3-beta hydroxysteroid dehydrogenase deficiency
   4. 5 alpha reductase deficiency

*Facilitators: While nonclassic CAH from A, B or C may explain the presentation of the patient in the vignette, 46 XY chromosome result of karyotype and the elevation in Test/DHT suggests the most likely etiology of this child is 5 alpha reductase deficiency. These patients have a potential for typical fertility and often virilize at puberty. Further, even if raised as girl before puberty, they often identify themselves as men after puberty finishes. 17-beta hydroxysteroid dehydrogenase deficiency is another disorder that can present similarly with features of androgen deficiency or a complete female phenotype in a 46 XY child.*

1. An 11-year old child raised as a girl is noted to have clitoromegaly (Prader 3), a palpable swelling in the inguinal canal and has pubic hair. Chromosomes show 46, XY pattern. Of the following the most likely diagnosis for this patient is:
   1. 21 hydroxylase deficiency
   2. 11-beta hydroxylase deficiency
   3. Mixed gonadal dysgenesis
   4. Partial androgen insensitivity

*Facilitators: Often, establishing etiology of 46XY DSD can be harder than for a 46 XX DSD. A patient with 21 hydroxylase deficiency or 11-beta hydroxylase deficiency and 46XY karyotype will not have atypical genitalia. Androgen insensitivity is by far the most common etiology of 46 XY DSD. PAIS often can present with a variable phenotype of androgen deficiency as seen in the patient. On the other hand, CAIS does not present with atypical genitalia. MGD is a much less common presentation. Partial gonadal dysgenesis is a possible etiology.*
