# Supplementary material for: Increasing Knowledge and Self-Efficacy on Differences in Sex Development (DSD): A Team-Based Learning Activity for Pediatric Residents
Source: MedEdPORTAL. 2021 Feb 23;17:11105. doi: 10.15766/mep_2374-8265.11105 (PMC7901252; doi:10.15766/mep_2374-8265.11105)
Supplement: Supplementary file 1 — Team Materials List.docxPre-Post Assessment iRAT Response Form.docxTBL Activity Slides.pptxStudent RAT.docxFacilitator RAT.docxFacilitator Team Application Activity.docxStudent Team Application Activity.docxAdrenal Enzyme Pathway Diagram.docxPrader Scale Handout.docx [file mep_2374-8265.11105-s001.zip › G. Student Team Application Activity.docx]

ATTENTION, STUDENTS: If you are accessing this material BEFORE it is used in your course, please do NOT read this document prior to the class session. This activity is designed to lead you through a learning experience that reinforces your knowledge of the content. Early review or dissemination of this material to others will diminish the learning opportunity and be considered academic misconduct.

Differences in Sex Development (DSD):
Team-Based Learning Module for Pediatric Residents

**Team Application Activity**

**Case 1**: You are examining a full-term newborn born via vaginal delivery. Mom had good prenatal care and no complications before or during the delivery. She was advised that they are having a boy based on the prenatal ultrasound. Baby weighs 3 kg and is 50 cm in length. Vitals: HR 140/min, RR 40/min, T 98.4 F, O2 saturation: 98%. Baby has a typical general exam. On GU exam, you note that the baby has Prader stage 4 ‘phallus’ and no palpable gonads. There is labial rugation, hyperpigmentation, and posterior labial fusion. Nurse mentions that the parents have been asking to speak to the doctor about the baby’s genitalia, gender assignment and medical issues. You have a team of two medical students and a nurse practitioner with you on rounds.

1. The student asks what are the best practices regarding gender discussion, surgery and medical assessment in this situation. What will be your response?
   1. The baby’s gender assignment should be a girl since there are no palpable gonads, they should not worry about any medical problems since the baby is a healthy weight, surgery can be done in next few days and baby will grow up as a female. They should ask rest of their class to come see the baby in the afternoon as well since baby might have a rare condition.
   2. The baby’s gender assignment is a boy since there is a well-developed penis, they should not worry about any medical problems since the baby is a healthy weight and baby will grow up as a male. Surgery will not be needed. You emphasize that they should maintain confidentiality and privacy of the family and not discuss with others.
   3. There is insufficient information available to determine the baby’s gender and additional testing will be needed to identify if baby is at risk of medical health problems. The need for surgery will be determined by etiology, performed with the child’s assent and preferably be postponed until late adolescence. You emphasize that they should maintain confidentiality and privacy of the family and not discuss with others.
   4. There is insufficient information available to determine the baby’s gender and additional testing will need to be done to decide if baby is at risk of medical problems. The need for surgery will be determined once an etiology is known but should be done in first few years of life if needed. They should maintain confidentiality and privacy of the family and not discuss with others
2. What electrolyte pattern will be most consistent with this baby’s current status?
   1. Sodium 129 meq/L, potassium 7 meq/L, CO2 16.
   2. Sodium 129, Potassium 5, CO2 19.
   3. Sodium 140, Potassium 5, CO2 21.
   4. Sodium 135, Potassium 5, CO2 16.
3. Baby has labs drawn prior to discharge and returns for follow up at 5 days of life. Baby is noted to be 5% below birth weight. Parents report baby is feeding well and on general exam appears well hydrated. Rapid FISH test suggests there is no Y chromosome material. Of the following, what test results would be MOST LIKELY for the additional pending lab test?
   1. 17 hydroxypregnenolone 25 ng/dl
   2. 17 hydroxyprogesterone 25000 ng/dl
   3. 17 hydroxypregnenolone 25000 ng/dl
   4. 17 hydroxyprogesterone 25 ng/dl

**Case 2**: You are examining a 15-year old girl who presents to the office with concern for amenorrhea. She reports starting breast development at age 10 years. Patient has no chronic medical problems but she has been suffering from depression so taking an SSRI. She is not sexually active. Mom thinks that her depression is partly because she feels ‘less than her peers’. Family history reveals one of the aunts had infertility.

She weighs 56 kg and is 165 cm tall. Her vitals show HR 72/min, BP 100/60 mm Hg. Her general exam shows Tanner 4 breast development but sparse pubic hair. There is no clitoral enlargement or a palpable gonad. She is not comfortable with a more detailed genitourinary exam. While you are awaiting the results of laboratory testing, ultrasound of pelvis returns showing absence of uterus or other Mullerian structures.

1. Of the following, which is the most likely diagnosis?
2. 5-alpha reductase deficiency
3. Complete androgen insensitivity syndrome
4. 3-beta hydroxysteroid dehydrogenase deficiency
5. 11-beta hydroxylase deficiency
6. Of the following, in this patient, laboratory testing will likely reveal which of the following pattern?
   1. FSH 4IU/ml, Testosterone 30 ng/dl, Prolactin 15 ng/ml, chromosome 46, XX
   2. FSH 2.5 IU/ml, Testosterone 300 ng/dl, prolactin 15 ng/ml, chromosome 46, XY
   3. FSH 40 IU/ml, Testosterone 30 ng/dl, prolactin 10 ng/ml, chromosome 46, XX
   4. FSH 40 IU/ml, Testosterone 300 ng/dl, prolactin 10 ng/ml, chromosome 46, XY

1. You advise the parents to return for a follow up visit and have the child attend as well. The laboratory test confirms diagnosis of a difference of sex development. Parents request that the child not be informed about the results because it will be traumatic for the child. How will you approach this?
2. Since child is a minor and child will not need surgery, it is appropriate to not tell the child about the diagnosis until adulthood.
3. Since child is a minor and child will not need surgery, it is appropriate if parents want to first learn about the diagnosis and then see a counselor to share the diagnosis with the child.
4. The diagnosis may or may not require surgery, however, it is appropriate if parents want to first learn about the diagnosis and then see a counselor to share the diagnosis and information about surgery with the child.
5. The diagnosis would require urgent surgery, therefore, it is appropriate to tell the child and the parents right away.
