# Supplementary material for: Increasing Knowledge and Self-Efficacy on Differences in Sex Development (DSD): A Team-Based Learning Activity for Pediatric Residents
Source: MedEdPORTAL. 2021 Feb 23;17:11105. doi: 10.15766/mep_2374-8265.11105 (PMC7901252; doi:10.15766/mep_2374-8265.11105)
Supplement: Supplementary file 1 — Team Materials List.docxPre-Post Assessment iRAT Response Form.docxTBL Activity Slides.pptxStudent RAT.docxFacilitator RAT.docxFacilitator Team Application Activity.docxStudent Team Application Activity.docxAdrenal Enzyme Pathway Diagram.docxPrader Scale Handout.docx [file mep_2374-8265.11105-s001.zip › I. Prader Scale Handout.docx]

Prader Scale

The Prader Scale is a scoring system for grading the degrees of genital masculinization. It was created to grade the degree of virilization in children with CAH and 46 XX chromosomes, all of whom were assumed to be “girls.” In fact, it represents a continuum of virilization, and an individual on the continuum is not automatically a girl.

The Prader Scale starts at “0”, which describes an individual with typically female external structures, and ends at “5” which describes a completely virilized individual, with typical male external genital structures at birth with the labial/scrotal sac empty since there are no testicles). The image below shows the Prader Virilization Scores.

**Stage I:** clitoromegaly without labial fusion

**Stage II:** clitoromegaly and posterior labial fusion

**Stage III:** greater degree of clitoromegaly, single perineal urogenital orifice, and almost complete labial fusion

**Stage IV:** increasingly phallic clitoris, urethra-like urogenital sinus at base of clitoris, and complete labial fusion

**Stage V:** penile clitoris, urethral meatus at tip of phallus, and scrotum-like labia (appear like males without palpable gonads)

| **Typical Female** | **I** | **II** | **III** | **IV** | **V** | **Typical Male** |
| --- | --- | --- | --- | --- | --- | --- |


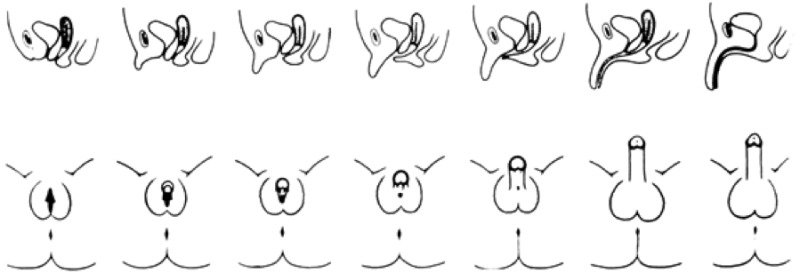


Image by MDText.com, Inc., retrieved from: [https://www.ncbi.nlm.nih.gov/books/NBK278953/figure/congn-adren-hyprplsa_f_congn-adren-hyprplsa_etx-ped-ch8-fig2](https://www.ncbi.nlm.nih.gov/books/NBK278953/figure/congn-adren-hyprplsa_f_congn-adren-hyprplsa_etx-ped-ch8-fig2/) on June 8, 2020. Image Used with permission. Modified to add labels for each stage.
