# Supplementary figures and images for: Increasing Knowledge and Self-Efficacy on Differences in Sex Development (DSD): A Team-Based Learning Activity for Pediatric Residents
Source: MedEdPORTAL. 2021 Feb 23;17:11105. doi: 10.15766/mep_2374-8265.11105 (PMC7901252; doi:10.15766/mep_2374-8265.11105)

The Adrenal Steroidogenesis Pathway


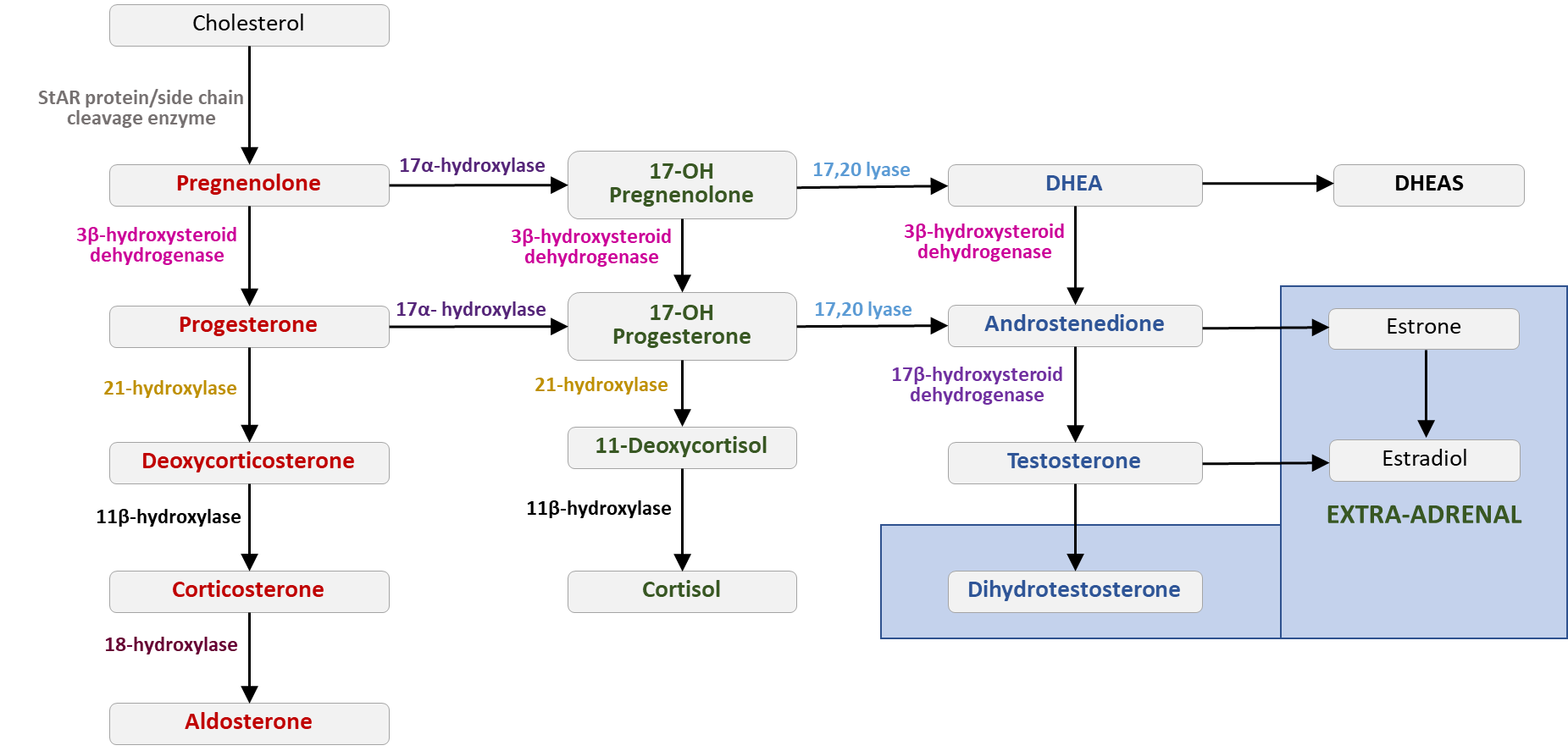


Image is author owned.

Supplement: Supplementary file 1 — Team Materials List.docxPre-Post Assessment iRAT Response Form.docxTBL Activity Slides.pptxStudent RAT.docxFacilitator RAT.docxFacilitator Team Application Activity.docxStudent Team Application Activity.docxAdrenal Enzyme Pathway Diagram.docxPrader Scale Handout.docx [file mep_2374-8265.11105-s001.zip › H. Adrenal Enzyme Pathway Diagram.docx]
